# Testing the genomic overlap between intraspecific mating traits and interspecific mating barriers

**DOI:** 10.1093/evlett/qrae042

**Published:** 2024-08-14

**Authors:** Leeban H Yusuf, Sonia Pascoal, Peter A Moran, Nathan W Bailey

**Affiliations:** Centre for Biological Diversity, School of Biology, University of St Andrews, Fife, United Kingdom; Department of Haematology, University of Cambridge, Cambridge, United Kingdom; A-LIFE, Section Ecology & Evolution, Vrije Universiteit Amsterdam, Amsterdam, The Netherlands; Centre for Biological Diversity, School of Biology, University of St Andrews, Fife, United Kingdom

**Keywords:** barrier loci, genotype–phenotype, reproductive isolation, sexual selection, speciation genomics

## Abstract

Differences in interspecific mating traits, such as male sexual signals and female preferences, often evolve quickly as initial barriers to gene flow between nascent lineages, and they may also strengthen such barriers during secondary contact via reinforcement. However, it is an open question whether loci contributing to intraspecific variation in sexual traits are co-opted during the formation and strengthening of mating barriers between species. To test this, we used a population genomics approach in natural populations of Australian cricket sister species that overlap in a contact zone: *Teleogryllus oceanicus* and *Teleogryllus commodus.* First, we identified loci associated with intraspecific variation in *T. oceanicus* mating signals: advertisement song and cuticular hydrocarbon (CHC) pheromones. We then separately identified candidate interspecific barrier loci between the species. Genes showing elevated allelic divergence between species were enriched for neurological functions, indicating potential behavioral rewiring. Only two CHC-associated genes overlapped with these interspecific candidate barrier loci, and intraspecific CHC loci showed signatures of being under strong selective constraints between species. In contrast, 10 intraspecific song-associated genes showed high genetic differentiation between *T. commodus* and *T. oceanicus*, and 2 had signals of high genomic divergence. The overall lack of shared loci in intra vs. interspecific comparisons of mating trait and candidate barrier loci is consistent with limited co-option of the genetic architecture of interspecific mating signals during the establishment and maintenance of reproductive isolation.

## Introduction

Determining whether and how sexual selection leads to speciation is a longstanding goal of speciation genomics, but it is also one of the most experimentally difficult (Mendelson & Safran, 2021; Ritchie, 2007; Servedio & Boughman, 2017). Evaluation of this idea requires identifying whether loci that cause intraspecific differences in secondary sexual traits also contribute to the origin, elaboration, or maintenance of interspecific reproductive barriers (Wilkinson et al., 2015). Such information is necessary to separate any signal of sexual selection during speciation from that caused by natural selection or neutral evolution, both of which can also act on sexual traits (Servedio & Boughman, 2017). Although theoretical and empirical studies have described ecological conditions favoring speciation via sexual selection (Kirkpatrick & Ravigné, 2002; Ritchie, 2007; Servedio & Boughman, 2017), few studies have identified loci underlying sexual traits and examined the forces acting on them during species divergence.

**Figure 1.**
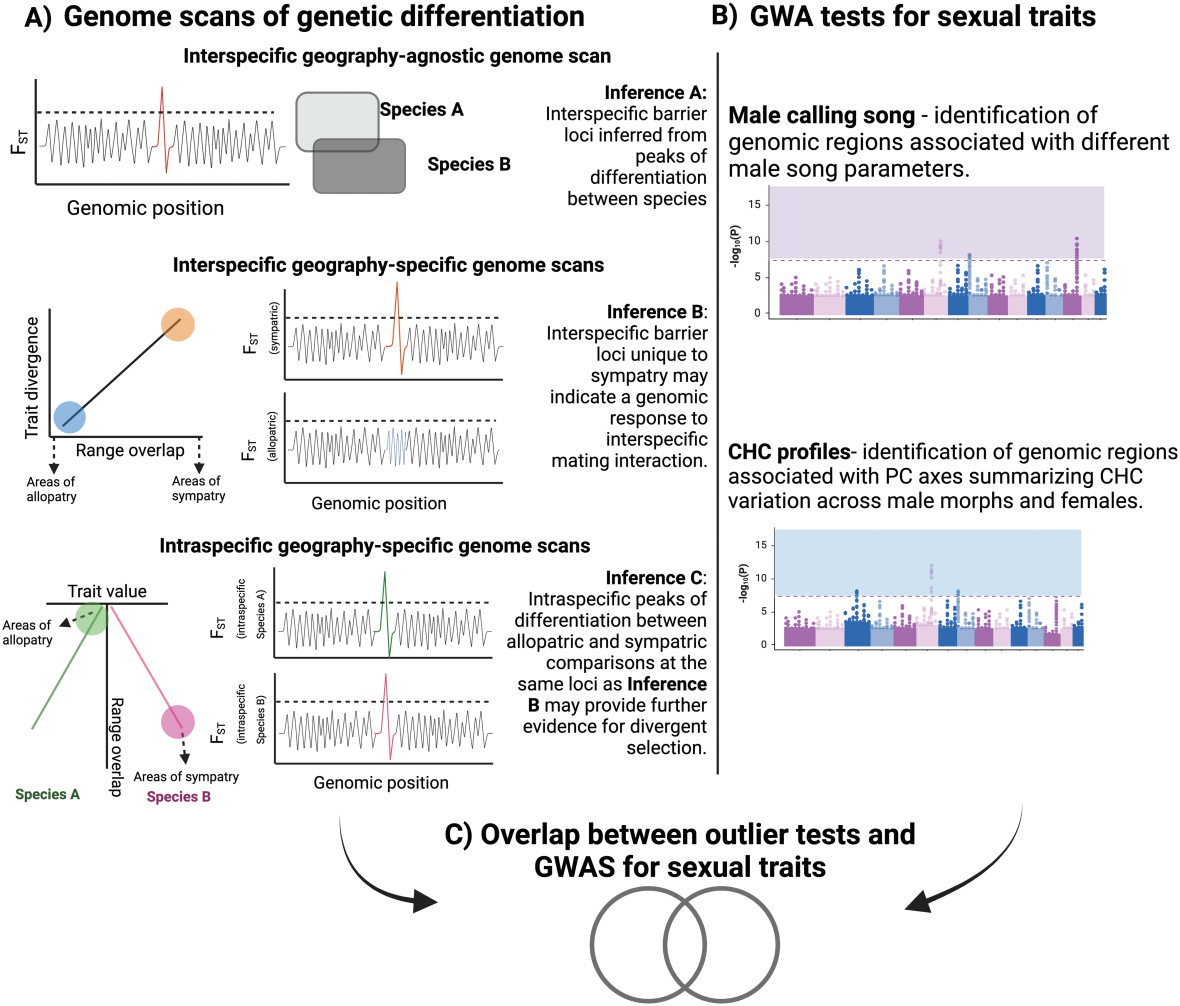
Combinatorial approach for detecting sexually selected loci implicated in reproductive isolation between *T. oceanicus* and *T. commodus*. (A) Genome outlier scans for putative barrier loci using genetic differentiation in three comparisons: between species only (top row), between species in different spatial contexts (middle row), and within species in different spatial contexts (bottom row). Contrasts between loci identified in each enable inference about genes implicated in species divergence versus reproductive character displacement. (B) Genome-wide association tests for sexual traits within species. (C) Testing for an overlap may result in no candidates being identified (a null expectation). Overlapping genomic regions found in both approaches may be promising candidate premating candidate barrier loci.

An effective way to study the link between the genetic basis of reproductive isolation and sexual traits is to test whether sexually selected loci overlap with candidate barrier loci (Brand et al., 2020; Turbek et al., 2021). Figure 1 illustrates an experimental approach to determine (a) whether any overlap between loci implicated in intrasexual selection and implicated in interspecific mating barriers exists and then (b) interrogate the evolutionary and geographical contexts under which these candidate loci have evolved. The approach combines intraspecific genotype–phenotype association tests with interspecific genome scans for divergent selection, the latter capitalizing on combined effects of local selection and gene flow—which generate identifiable heterogeneity in genomic landscapes—to search for barriers to gene flow (Ravinet et al., 2017). Compared with genomic investigations of ecological differentiation (Seehausen et al., 2014), few causal loci underlying variable mating phenotypes between species have been identified. Nevertheless, those studies that have detected candidate loci using genotype–phenotype associations have most often identified oligogenic architectures for mating signals and preference between incipient species (Brand et al., 2020; Ding et al., 2016; Merrill et al., 2019; Turbek et al., 2021; Unbehend et al., 2021). Regions underlying sexual trait differences that are also implicated in reproductive isolation are expected to show heightened genetic differentiation compared to the genomic background (Wilkinson et al., 2015). Furthermore, characterizing the potential demographic history of closely related species and evaluating gene flow between them can help to identify genomic signals consistent with character displacement. An important distinction can then be made to identify loci implicated in sexual differences arising from reinforcement to avoid maladaptive hybridization during secondary contact: these are predicted to show enhanced genetic differentiation in sympatry compared to allopatry (Garner et al., 2018).

In this study, we used genotype–phenotype association tests and population genomic analyses across a geographical transect containing a contact zone of Australian field cricket sister taxa (*Teleogryllus* spp.) to test the genomic overlap between intraspecific mating traits and candidate interspecific barrier loci. *Teleogryllus oceanicus* and *T. commodus* are a classic system for studying acoustic sexual signaling and do not show evidence of ecological differentiation or habitat preference despite contact in sympatric regions of their Australian ranges (Otte & Alexander, 1983). They differ in long-range calling song used for mate location and female choice (Hill et al., 1972; Bailey & Macleod, 2014), and recent behavioral analyses suggest discrimination by both sexes resulting in robust, symmetrical behavioral isolation between species (Moran et al., 2018). Though the species readily hybridize in the laboratory, population genetic analysis along a 2,500 km latitudinal transect of their Australian range showed no evidence of recent hybridization in the wild (Moran et al., 2018).

Using two extensive genomic datasets (Moran et al., 2018; Pascoal et al., 2020) and a new, high-quality chromosome-contiguous genome assembly for *T. oceanicus* (Zhang et al., 2024), we characterized the genetic architecture of male calling song and male contact pheromones (cuticular hydrocarbons: CHCs). We then identified loci associated with patterns of divergent selection across 16 sympatric and allopatric populations of the two species. Elevated differentiation in sympatry and inferred historical gene flow provided evidence consistent with reinforcement or asymmetric demographic changes in the contact zone. Loci related to intraspecific variation in CHCs and male advertisement songs were localized throughout the genome, and more song loci overlapped with putative interspecific barrier loci than loci associated with CHCs. The findings imply limited co-option of loci underlying intraspecific mating traits during the formation or maintenance of interspecific mating barriers. However, those loci that are co-opted may underpin sexual trait modalities that are less constrained by ecological factors and play a more prominent role in speciation.

## Methods

### Sampling, read mapping, and filtering

**Figure 2.**
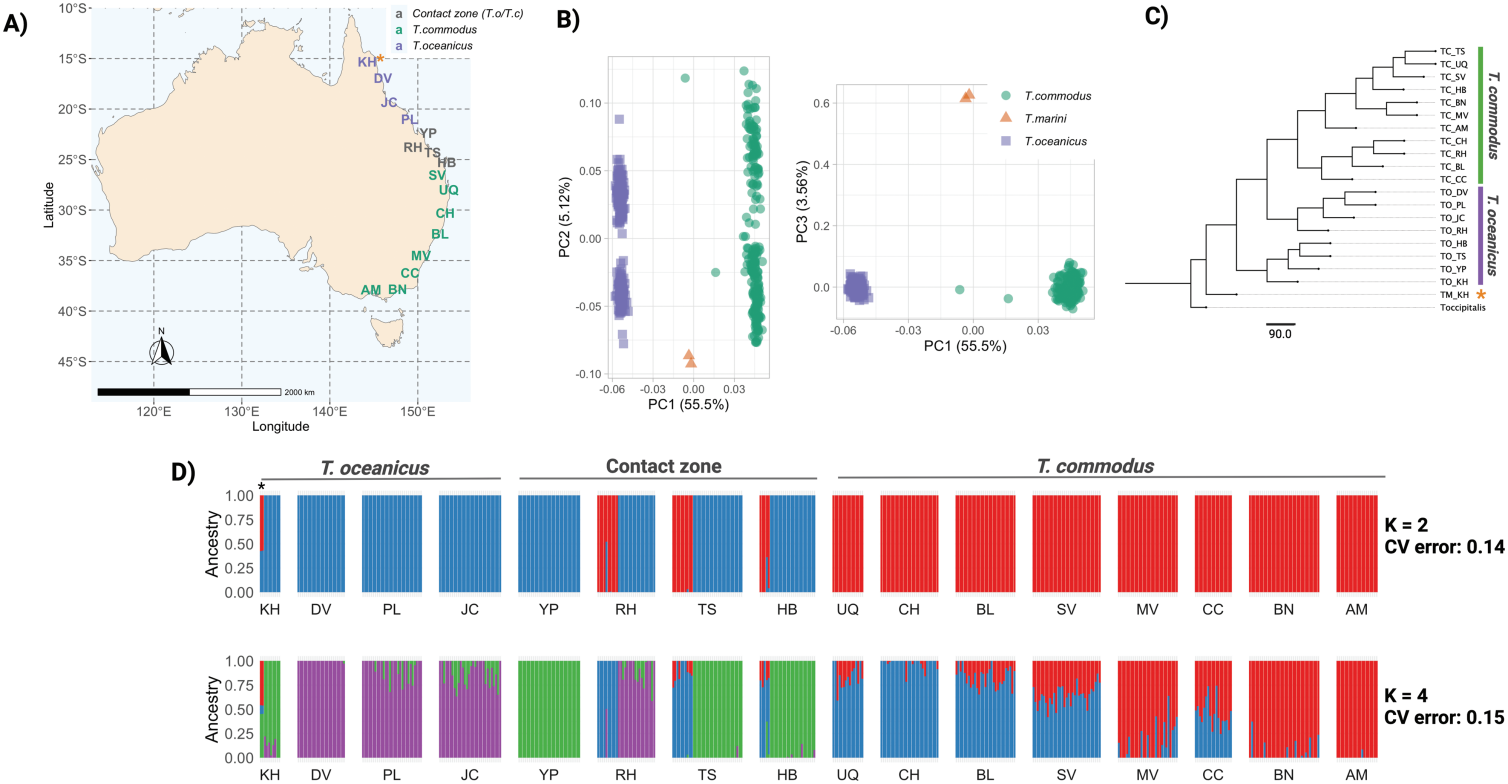
Geographic sampling scheme and population structure. (A) *T. oceanicus*, *T. commodus* and *T. marini* population sampled by Moran et al (2018) and used in this study. Colors denote allopatric and sympatric populations of *T. commodus* and *T. oceanicus*. Asterisks denotes population (KH) where *T. marini* was found. (B) PCAs of genetic variation across all samples and 4,163 linkage-pruned SNPs. (C) Multispecies coalescent tree of populations and species, with all branches showing maximal branch support in SVDquartets (100%). (D) Inferred admixture fractions for allopatric and sympatric populations under *K* = 2 (top panel) and *K* = 4 (bottom panel). Asterisk denotes *T. marini* samples, which show mixed ancestry.

To characterize patterns of genetic variation across the genome and understand the speciation histories of *T. commodus* and *T. oceanicus*, we used genomic data from eight allopatric populations of *T. commodus* in southern Australia, and four allopatric populations of *T. oceanicus* in northern Australia (Figure 2A) (Moran et al., 2018). We also used genomic data from individuals of both species sampled within four sympatric populations spanning a contact zone in the middle of the geographical transect. In one of the northern Queensland populations, we obtained samples for a third Australian species in the genus, *T. marini,* representing an additional contact zone containing *T. oceanicus.* For *T. commodus* and *T. oceanicus*, between 10 and 34 individuals were sampled per population; detailed sample information can be found in Supplementary Table 1. In total, raw, demultiplexed, reduced representation sequence data (RAD-seq) for 16 populations of allopatric and sympatric *T. oceanicus*, *T. commodus*, and *T. marini* individuals of both sexes were used. These raw reads were previously published by Moran et al. (2018).

Reads were trimmed using fastp v.0.20.1 (Chen et al., 2018) with default parameters, then mapped to a *T. oceanicus* reference genome (Zhang et al., 2024) using BWA mem (Li, 2013). We used samtools (v. 1.11) (Li et al., 2009) to index, sort, mark, and remove duplicate reads from each sample and Picard (Institute, 2019) to add read groups to each sample. Variant calling was performed using bcftools mpileup and call function with the following parameters: -C 50/ -m -f GQ,GP. Variants were filtered for minimum depth (10), max missingness (0.5), minimum quality score (30), minor allele count (3), and individuals with missing genotypes at half or more of all variable sites were removed. In total, 1,451,414 variants were called, and 89,045 were retained following filtering. We found SNPs in 0.004% of the ca. 2.03 Gb genome and 14.20% of protein-coding genes (3,012 out of a total of 21,211) were covered by at least one SNP. Additionally, after removing individuals with more than 50% missing sites, we retained 417 out of 465 individuals for analysis.

### Population structure and phylogenetic reconstruction

We used PLINK (v1.90b) to assess the population structure for all retained samples. To assess only independent, nonlinked genetic variation, we further filtered our dataset of 89,045 biallelic SNPs down to 4,163 using the following parameters in PLINK: “--indep-pairwise 50 10 0.01” to remove variants with *r*^2^ larger than 0.01 every 10 bp in 50 kb windows. We reconstructed phylogenetic relationships between allopatric and sympatric populations of *T. commodus*, *T. oceanicus*, and *T. marini* using *T. occipitalis*, an Asian congener, as an outgroup. This involved mapping whole-genome sequence data of *T. occipitalis* from Kataoka et al. (2020) to our reference *T. oceanicus* genome using BWA mem (Li, 2013) as above. We used samtools (v. 1.11) (Li et al., 2009) to index, sort, mark, and remove duplicate reads. Variants were called and filtered for minimum depth (10) and minimum quality score (30). We merged filtered variants of *T. occipitalis* and *T. oceanicus*, *T. commodus*, and *T. marini* to produce a comprehensive SNP dataset of 94,993 variants (i.e., a separate set of SNPs from the set of 89,045 obtained for population genomic analyses above) for gene flow and phylogenetic inference. With this, we inferred a species tree using multispecies coalescent inference via SVDquartets and PAUP (v. 4a) (Chifman & Kubatko, 2014).

To understand population structure and contemporary patterns of hybridization, we inferred ancestry proportions for samples across all 16 populations using the likelihood-model approach in ADMIXTURE (Alexander et al., 2009), with *K* genetic clusters ranging from 1 to 4. We used all 89,045 SNPs in our filtered dataset described above. We converted data from VCF format to PLINK format using PLINK (v1.90b) and ran three iterations of ADMIXTURE for models up to *K* = 4. We evaluated the fit of the four models using the cross-validation procedure.

### Demographic modeling and inference of gene flow

To estimate the prevalence of introgression between *T. commodus* and *T. oceanicus* and the spatial context of gene flow, we estimated Patterson’s *D* statistic in *Dsuite* (Malinsky et al., 2020) across the set of 94,993 biallelic SNPs described above. Specifically, we calculated D and f4 statistics to estimate gene flow between *T. oceanicus* (P2) and *T. commodus* (P3) by grouping populations by species and using *T. occipitalis* as an outgroup and *T. marini* (P1) as a derived species. Separately, we assessed D and f4 statistics across all possible combinations of populations in trios, resulting in 1,141 comparisons of three different site patterns (ABBA, BBAA, BABA). Standard jackknife procedure across blocks was used to assess significance, with *p*-values corrected using Benjamini & Hochberg false-discovery rate correction (Benjamini & Hochberg, 1995). *D*-statistics were considered significant only when *Z*-scores were larger than three, remained significant following multiple correction (*p* < 0.05), and where *D* was larger than 0.05.

To accompany estimates of gene flow, we inferred the likely demographic history of speciation between *T. commodus* and *T. oceanicus* using *dadi* (Gutenkunst et al., 2009) and a modular *dadi* pipeline (Portik et al., 2017). We converted our filtered VCF containing 89,045 biallelic SNPs for population genomics to a joint site-frequency spectrum using easySFS (https://github.com/isaacovercast/easySFS). The SFS was projected down to 168 and 242 for *T. oceanicus* and *T. commodus*, respectively. Consecutive optimization rounds were performed for each model, with four multiple replicates (replicates = 10, 20, 30, 40) in each round. The best-scoring parameter estimates (based on log-likelihood) were used to seed searches in the following round. Parameters were optimized using the Nelder–Mead method.

We considered a series of simple demographic scenarios (Supplementary Figure 1): (1) a “no migration” model where species divergence occurred with no gene flow (included three parameters: divergence time, *N*_*e*_ for species *A*, *N*_*e*_ for species B), (2) a “symmetric migration” model where species divergence was followed by a continuous period of symmetric gene flow (included a fourth parameter of constant gene flow), (3) an “ancestral migration” model where divergence occurred with gene flow and ceased at some time point (included a fifth parameter of a second time denoting the end of gene flow), and (4) a “secondary contact” model where symmetric migration occurs after some timepoint (included a fifth parameter of a second time denoting the beginning of gene flow).

### Population genetic summary statistics

We calculated population genetic summary statistics across the genome to understand genetic differentiation and divergence between allopatric and sympatric populations of *T. oceanicus* and *T. commodus.* We first estimated site-wise Weir and Cockerham’s FST using VCFtools (v0.1.16) (Danecek et al., 2011) for five different comparisons using subsets of our filtered SNP dataset: (1) between all *T. commodus* and *T. oceanicus* individuals, (2) between only allopatric populations of *T. commodus* and *T. oceanicus* individuals, (3) between only sympatric populations of *T. commodus* and *T. oceanicus* individuals, (4) between allopatric and sympatric populations of *T. commodus*, and (5) between allopatric and sympatric populations of *T. oceanicus*.

Separately, we calculated between-species FST, sequence divergence (d_XY_), and nucleotide diversity (π) by first recalling variants alongside invariant sites. We filtered sites in the VCF as described above (minimum depth (10), max missingness (0.5), minimum quality score (30), minor allele count (3), and individuals with missing genotypes at half of all variable sites were removed). We estimated d_XY_ and π using Pixy (Korunes & Samuk, 2021) in 10 kb windows with 10 kb step sizes. We note that RAD-seq is often associated with well-known challenges related to accurately surveying genetic diversity throughout the genome. This is likely ameliorated by our dense sampling across the geographic transect. We calculated d_XY_ between *T. commodus* and *T. oceanicus* individuals and d_XY_ between both species in sympatry and allopatry separately. Functional identification was performed using custom scripts which primarily involved blasting coding sequences against UniProt databases. Gene set enrichment analysis was performed in STRING (Szklarczyk et al., 2015) for between-species F_ST_ outlier genes. Specifically, outlier gene names were passed to STRING, which performed hypergeometric testing against a statistical background of all *D. melanogaster* protein-coding genes. *p*-values for the enrichment tests were corrected for multiple testing using the Benjamini–Hochberg procedure (Benjamini & Hochberg, 1995). The strength of the enrichment is calculated as log10 of the number of observed genes divided by the expected number of genes annotated within a given functional category.

### Genetic associations for CHC and male calling song traits

We performed genotype–phenotype association tests for two sexually selected traits—male calling song and cuticular hydrocarbon pheromone profiles—involved in species discrimination. These two phenotypes were scored in *Teleogryllus oceanicus* only due to limitations of laboratory stock. This precluded us from testing genetic associations with traits directly involved in interspecific mating discrimination; however, it enabled us to test whether loci implicated in intraspecific mating variation show enhanced divergence in targeted interspecific comparisons, providing a powerful and unusual test of the connection between intraspecific sexual selection and interspecific divergence. We used RAD-seq data for F_3_ offspring previously produced from two mapping families described in Pascoal et al. (2020). Families were set up by initially crossing a sire from a Kauai, Hawaii stock line and a virgin dam from a Daintree, Australia stock line. These had been selected to maximize intraspecific variation in sexually selected traits of interest (Pascoal et al., 2017). Subsequently, F1 full-sib matings and then F2 full-sib matings were performed to produce 10 F3 mapping families consisting of 192 females and 199 males (of which 113 had “normal-wing” venation which produces audible acoustic signals, and 86 had silent “flatwing” venation owing to an adaptive male-silencing trait that is polymorphic in Hawaii) (Pascoal et al., 2014, 2020; Zuk et al., 2006).

#### Male calling song phenotyping

Following Moran et al. (2020), we scored 14 parameters of *T. oceanicus* male calling song in the F3 mapping population, which we considered as independent, continuous phenotypic variables. The parameters can be found in Supplementary Table 2 and all but carrier frequency are related to duration and temporal patterning of chirps within the standard, two-component *T. oceanicus* song phrase. It is important to note that all three *Teleogryllus* species have similar two-component songs consisting of a higher-amplitude trill followed by lower-amplitude chirps but with different frequency and temporal features, lending plausibility to the hypothesis that loci underlying intraspecific song variation in *T. oceanicus* might also be under selection during interspecific encounters. Male calling songs were recorded using a Sennheiser ME66 microphone under a red light between 22 °C and 25 °C (with the exception of a single individual recorded at 19 °C). Five song phrases from a continuous recording of each male’s calling song were manually analyzed using Sony SoundForge (v.7.0a). Each parameter was then averaged within each individual to obtain a set of 14 calling song traits for association mapping. We used song data from 84 mapping individuals after retaining only those that were successfully phenotyped as well as genotyped.

#### Cuticular hydrocarbon phenotyping

CHC profiles for mapping individuals were obtained from previously published data by Pascoal et al. (2020). Methods are fully described in Pascoal et al. (2016, 2020), but to summarize, individually flash-frozen *T. oceanicus* crickets were immersed in HPLC-grade hexane (Fisher Scientific) for 5 min to remove long-chain waxy hydrocarbons that coat the insect cuticle. The resulting material was analyzed with an internal pentadecane standard on an Agilent 7890 gas chromatograph linked to an Agilent 5975B mass spectrometer. Instrument run conditions were previously optimized for *T. oceanicus* (Pascoal et al., 2016). Mass spectrometry was performed using a C_7_–C_40_ alkane standard to enable the calculation of peak retention indices (Supplementary Table 3). For each sample, 26 peaks were integrated and quantified using MSD CHEMSTATION (v.E.02.00.493). The resulting peak abundances were entered into a principal components analysis (PCA) to reduce dimensionality for association mapping. Both sexes, including two polymorphic male forms (silent “flatwing” and singing “normal-wing”) present in the Hawaiian mapping population, were combined in the PCA because we were primarily interested in the genetic architecture and loci associated with major sources of intraspecific CHC variation irrespective of sex or morph, as CHCs of both sexes are subject to sexual selection in this species (Thomas & Simmons, 2009, 2010). Accounting for individuals that were successfully phenotyped as well as genotyped, PCs 1–4 of 328 individuals entered the association mapping pipeline described below. CHCs appear to be perceived as a complex, multicomponent “bouquet” under strong multivariate selection in *T. commodus* and *T. oceanicus* (Thomas & Simmons, 2009, 2010), but unlike in *Drosophila* there is no evidence that only one or a few individual peaks play a disproportionate role in mate choice. Since we could not exclude that possibility, however, we checked that our dimensionality-reduction procedure did not qualitatively affect the inferences of our experiment by selecting the three highest-loading peaks (peaks 4, 8, and 9) and running the same analyses for each individually.

#### Association analyses

The primary aim of our association analyses was to identify large-effect candidate loci associated with intraspecific variation in sexual traits. We conducted genotype–phenotype association analyses using a Wald test in GEMMA (v0.98.3) (Zhou & Stephens, 2012, 2014). For this, we fitted univariate linear mixed models to test for the association between each song parameter and SNP genotypes, accounting for population structure by jointly incorporating an inter-individual relatedness matrix along with individual mass and individual pronotum lengths as covariates. For CHCs, we fit a multivariate linear mixed model to test for an association between genetic variation and all four PC axes, capturing most CHC variation (63.15%) among individuals. Here, we decide to use PCA to identify relevant genes based on previous successful attempts to study the genetic basis of CHC production in *D. melanogaster* using a similar dimensionality reduction approach (Dembeck et al., 2015). More PC axes could not be included in the multivariate model due to computational limitations in GEMMA. In both analyses, we aimed to identify loci associated with intraspecific variation in song and CHCs and variation that might be important in maintaining isolation between *T. oceanicus* and *T. commodus*. However, genetic variants associated with CHCs and male calling songs are restricted to intraspecific differences between *T. oceanicus* individuals, such that unique features of song and CHC profiles that might be specifically found in *T. commodus* are not studied here. To assess significance, we adjusted *p*-values using a Bonferroni–Hochberg procedure with a significance threshold of corrected *p *< 0.05. We note that since we perform separate association tests for each component of the song, the chance of detecting false positives across association tests increases for each test where there are any significant associations. To visualize these results, we plotted the log-transformed corrected *p*-values and switched their sign. Finally, we tested whether significantly associated song and CHC SNPs are implicated in divergence between *T. oceanicus* and *T. commodus* by first assessing the overlap between 99th quantile F_ST_ and d_XY_ 10 Kb windows in our interspecific genome outlier scans with song and CHC-associated SNPs. Subsequently, we compared mean F_ST_ and d_XY_ 10 Kb windows for song and CHC-associated regions with genome-wide mean F_ST_ and d_XY._ We tested differences in means using two-sample *t*-tests.

We calculated the integrated haplotype score (iHS) to test for evidence of selective sweeps in two candidate regions associated with short-chirp inter-chirp intervals. We first phased our filtered SNP dataset using SHAPEIT4 with default parameters (Delaneau et al., 2019). We then used the R package *rehh* to filter data for minor allele frequency (minimum MAF < 0.05) and run iHS scans for *T. commodus* and *T. oceanicus* separately. To identify genes implicated in song divergence, we looked for genes showing extreme iHS values (99th percentile) and containing an SNP with significant association to a song parameter (particularly, the “short-chirp inter-chirp interval” parameter; see Supplementary Table 2) using the *bedtools intersect* function.

#### Genotype-environment associations

To infer whether mating traits may potentially be under ecological selection or if local adaptation across environmental gradients confounds signals of divergent selection between *T. commodus* and *T. oceanicus*, we performed genotype-environment association (GEA) scans. GEA approaches work by testing whether SNP allele frequencies are significantly associated with relevant environmental variables after correcting for population structure. Here, since *T. oceanicus* and *T. commodus* populations show a clear latitudinal gradient, we utilize the latitude and longitude of each population as environmental variables. We chose latitude and longitude to account for any differences between population and species in important environmental factors which could include day-length, seasonality, temperature, and precipitation, all of which are expected to covary with geography. Ecological variables may also vary with geography, though we have observed no systematic difference in vegetation and land cover between the *Teleogryllus* species studied here. Similar analyses have been performed with latitude and longitude as proxies for other environmental variables (Gain & François, 2021). Latent factor mixed models are univariate models that test the association between each SNP and an environmental variable and are among the most powerful methods to detect GEAs (Gain & François, 2021). These approaches require estimation of background structure via inference of the number of latent factors (*K*) in the dataset. We used sparse nonnegative matrix factorization (snmf) to estimate the number of latent factors and chose an optimal *K* (*K* = 6) based on which *K* had the lowest cross-entropy criterion score, using the *R* package LEA (Frichot & François, 2015; Frichot et al., 2014).

LFMM was performed using a ridge regression on the full set of 89,045 SNPs and parameterized using a *K* = 6 via the *R* package *lfmm* (Caye et al., 2019; Frichot et al., 2013)*. p*-values were calibrated for each SNP using a genomic inflation score. We conservatively characterized loci as “environmentally-associated” if calibrated *p*-values for both latitudinal and longitudinal association tests were *p* < 0.001. We tested the overlap between genes containing environmentally associated SNPs and genes containing song and CHC-associated SNPs.

## Results

### Population structure

Figure 2B shows genome-wide PCAs examining population genetic structure in *T. oceanicus* and *T. commodus*. The species were largely separated into two clusters based on PC1 (which explained 55.5% of the total variation). Both species show comparable intra-species variation along PC axes. This analysis also included samples from the third sister species, *T. marini*, which co-occurs in North Queensland with *T. oceanicus* and differs from *T. oceanicus* and *T. commodus* in calling song and coloration (Otte & Alexander, 1983; Moran et al., 2020). *T. marini* samples show extreme values on PC3 (explaining 3.5% of total variance) and are distinct from T. *oceanicus* and *T. commodus*.

The clustering of samples by species identity in the PCA was supported by multispecies coalescent phylogenetic analyses. However, whilst populations of *T. commodus* were monophyletic, *T. oceanicus* populations were not (Figure 2C). Our phylogenetic inference also supports the early divergence of *T. marini* followed by *T. oceanicus* and then *T. commodus* populations, suggesting north-to-south evolutionary splitting along the geographical transect. We found a very low, but nonzero, fraction of mixed-ancestry samples in the *T. oceanicus* and *T. commodus* contact zone, recapitulating findings from Moran et al. (2018b) (Figure 2D) and suggesting that hybridization in the field, while very rare, may occur. Additionally, ancestry proportions under *K* = 4 reveal a latitudinal gradient for *T. commodus*, but not in *T. oceanicus*.

### Demographic history and historic gene flow between *Teleogryllus* species

**Figure 3.**
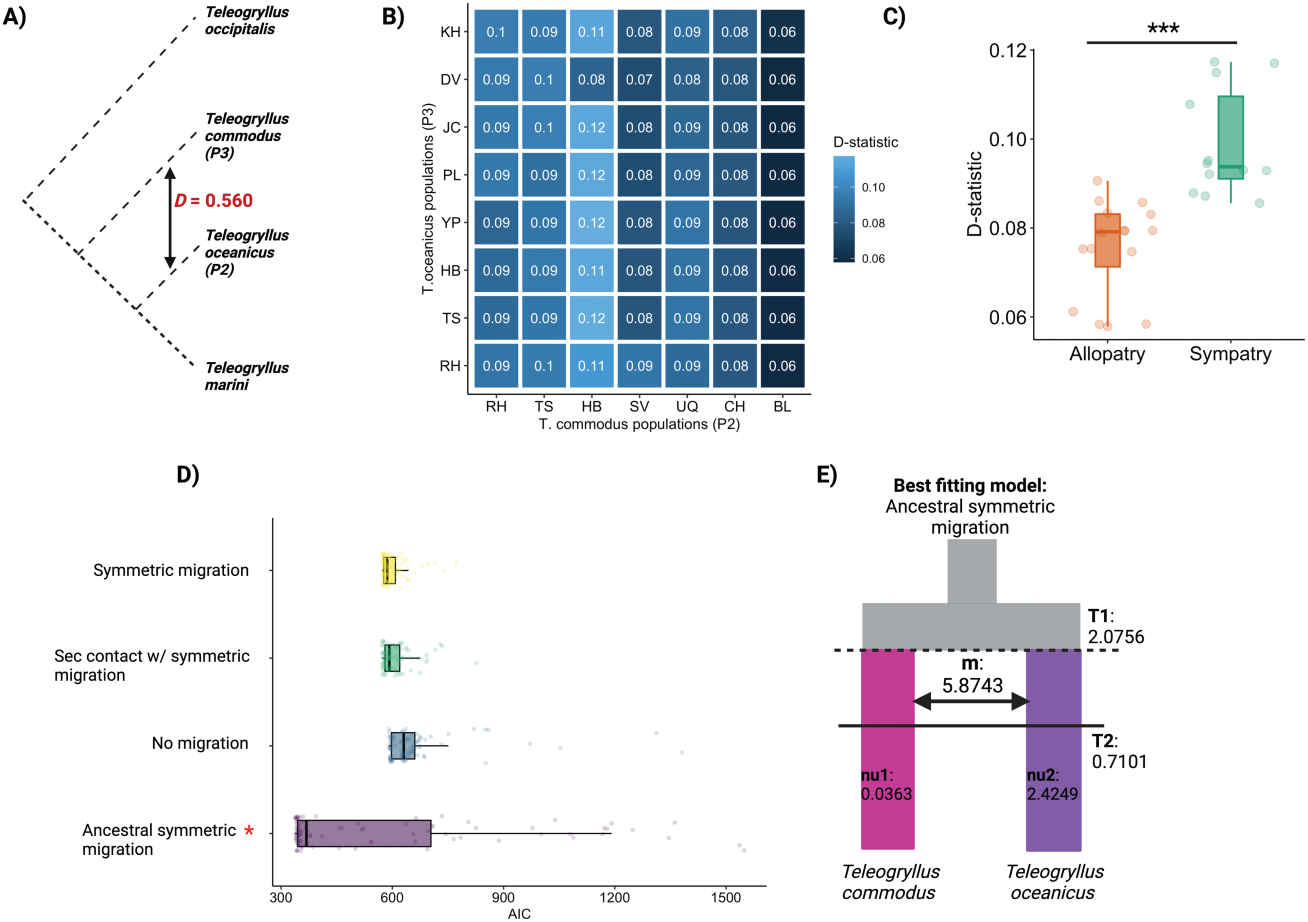
Population demography and historical gene flow. (A) Inferred gene flow (*D* statistic) between *T. oceanicus* and *T. commodus.* (B) Gene flow between *T. oceanicus* and *T. commodus* populations, with *T. occipitalis* as the outgroup. *D*-statistics were averaged for independent tests where P1 was permuted across all populations. Only comparisons with significant *p*-values (after multiple testing) and *Z*-scores < 3 are shown here. (C) Gene flow inference across allopatric and sympatric populations of *T. oceanicus* and *T. commodus.* Comparison of *D*-statistics for trios where P2 (*T. commodus*) and P3 (*T. oceanicus*) are allopatric or where both sister species are sympatric. Stars indicate level of significance: 0.0001 = ***, 0.001 = **, 0.01 = *, 0.05 =., >0.05 = ns. (D) Model fits for different species divergence scenarios with and without gene flow, using *dadi*. Each point shows AIC for an independent model-fitting run. Red asterisks show the best-fitting model after 50 optimized, independent runs. (E) Diagram showing the best-fitting model (divergence with ancestral symmetric migration between *T. oceanicus* and *T. commodus*) unscaled parameter estimates of T1 (initial divergence parameter), T2 (time of cessation of gene flow period parameter), m (symmetric migration), nu1 (population size of *T. commodus*) and nu2 (population size of *T. oceanicus*).

Whilst contemporary gene flow between *T. commodus* and *T. oceanicus* appears to be rare in contact zone populations, we detected significant historical gene flow between both species (Figure 3A), and significantly elevated *D*-values in contact zone populations compared to allopatric populations (Figure 3C). Whether significant trios (*t* = −5.32, *p *< 0.001) or nonsignificant trios (*t* = −2.36, *p *= 0.02) were considered did not alter the observation of stronger gene flow in contact zone populations. The best-fit demographic models supported a scenario of historical gene flow in sympatry that continued after gene flow in allopatry stopped. Interestingly, patterns of gene flow were consistent for all population comparisons but particularly consistent among *T. commodus* populations (Figure 3B). These results indicate that ancient hybridization events very early on in species divergence are likely responsible for correlated *D*-values observed between species, and sympatric *T. commodus* populations may have experienced more unidirectional gene flow from *T. oceanicus* than vice versa. This is consistent with two *T. commodus*-assigned individuals showing mixed ancestry in the contact zone (1B & 1D). To investigate this in more detail, we compared models of continuous, ancient, and recent migration in our demographic modeling framework. We found the best support for an ancient migration model with symmetric isolation based on consistently low AIC scores across optimization rounds, particularly in the final model optimization round, where model convergence is expected (Figure 3D and E and Supplementary Figures 5 and 7).

### Pronounced, asymmetrical genomic differentiation in sympatry

**Figure 4.**
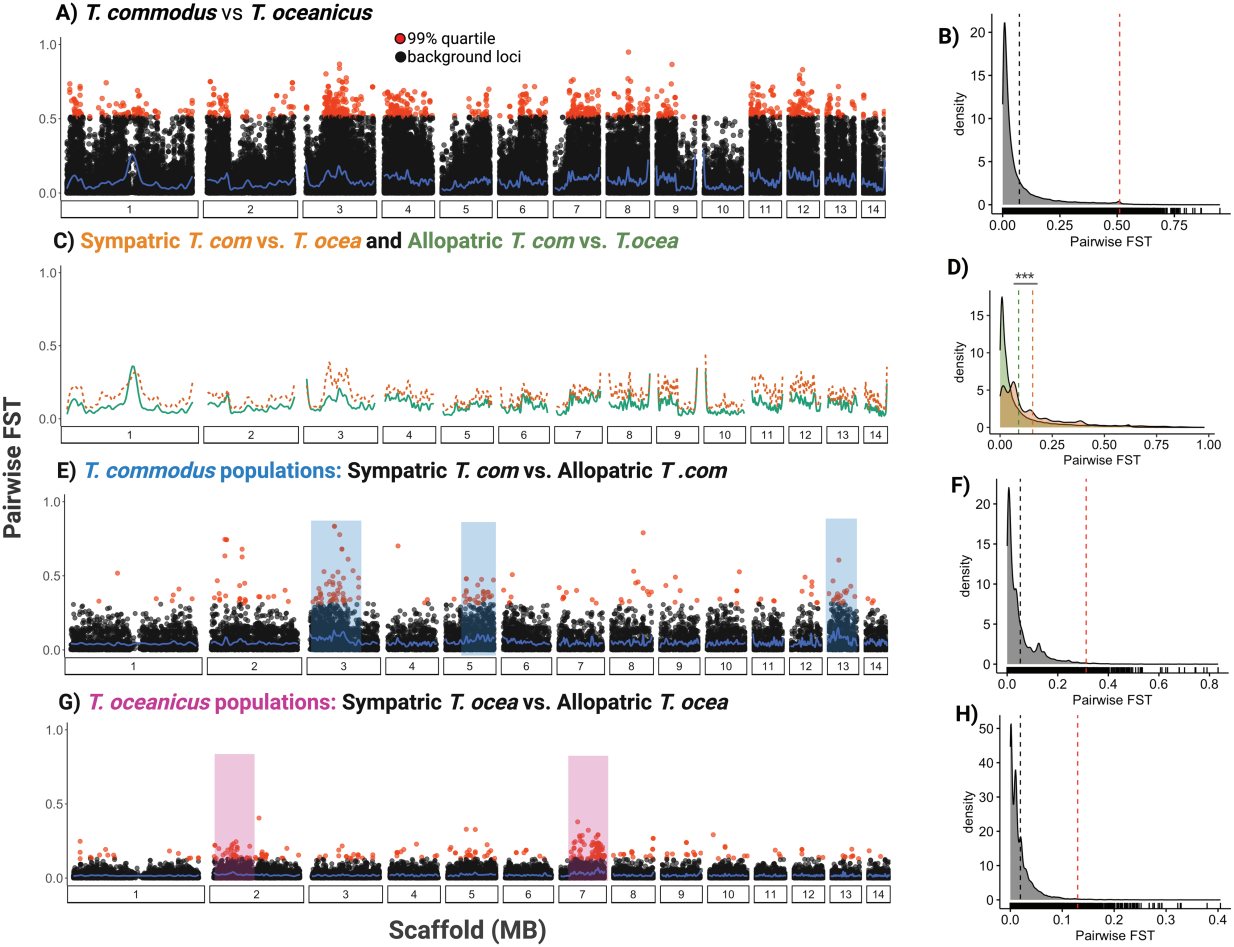
Population-pairwise F_ST_ between and within *T. commodus* and *T. oceanicus*. (A) Site-wise F_ST_ between all *T. commodus* versus *T. oceanicus* population pairs. (B) Density of sites (short vertical black bars above the *x*-axis), distribution of their pairwise F_ST_ (shaded region), mean F_ST_ (dashed black line), and 99th quantile outlier mean (dashed red line). (C) Smoothed site-wise F_ST_ comparing sympatric *T. commodus* and *T. oceanicus* (dashed orange line) and allopatric *T. commodus* and *T. oceanicus* (solid green line). (D) Distributions of pairwise F_ST_ in sympatric (orange) and allopatric (green) comparisons. Stars indicate significance at *p* < 0.0001. (E) Site-wise F_ST_ between *T. commodus* populations sympatric with *T. oceanicus* versus allopatric with *T. oceanicus.* Blue shading indicates genomic regions enriched for outlier loci. (F) Distribution of pairwise F_ST_ with mean (black dashed line) and mean for 99th quantile outlier SNPs (dashed red line). (G) Site-wise F_ST_ between *T. oceanicus* populations sympatric with *T. commodus* versus those allopatric with *T. commodus*. (H) Distribution of pairwise F_ST_ showing mean (black dashed line) and mean for 99th quantile outlier SNPs (dashed red line). Pink shading indicates genomic regions enriched for outlier loci.

Average F_ST_ between species was 0.072, with significantly lower differentiation on the X chromosome (F_ST_ = 0.063) compared to the autosomes (F_ST_ = 0.074) (two-sample *t-test*: *t* = 11.02, *p *< 0.0001) (Supplementary Figure 2). Average F_ST_ between *T. commodus* and *T. oceanicus* in sympatry (F_ST_ = 0.156) was significantly higher than in allopatry (F_ST_ = 0.088) (two-sample *t-test*: *t* = −65.58, *p *< 0.0001) (Figure 4C and 4D). Heightened differentiation in the contact zone is attributable to greater divergence of *T. commodus* contact zone populations: average F_ST_ between sympatric *T. commodus* and allopatric *T. oceanicus* (F_ST_ = 0.155) replicated the considerable difference in average F_ST_ between sympatric populations of both species, but F_ST_ between sympatric populations of *T. oceanicus* compared to allopatric populations of *T. commodus* (F_ST_ = 0.0804) did not (see also Figure 4E and F compared to Figure 4G and H). Our results indicate that genomic landscapes of differentiation may be explained by asymmetric selection against gene flow or demographic shifts in the contact zone between *T. commodus* and *T. oceanicus*.

To identify genomic regions potentially involved in species divergence, we characterized F_ST_ outlier SNPs between *T. commodus* and *T. oceanicus*, and between sympatric and allopatric populations within the same species (intraspecies, interpopulation). We ran the latter procedure separately for both species. We found 4,457 (99th quantile) outlier SNPs between species out of 89,045 SNPs and identified 243 genes with functional annotations that overlapped with outlier SNPs. Apart from scaffold 10, outlier SNPs were distributed almost uniformly across the genome (Figure 4A and B). For these loci, a third of significant Gene Ontology terms relate to neural development (Supplementary Figure 3). To increase the robustness of inferences about potential genomic barriers to gene flow, we also calculated genome-wide d_XY_ in 10 kb windows and identified 651 outlier windows (d_XY_ > 0.0149). Only 43 genes were found to overlap with the 651 d_XY_ outlier windows. Using a combinatorial approach to identify the best-supported candidate gene set underlying species divergence, we examined the intersection of F_ST_ and d_XY_ outliers and found 84 shared outlier regions and 17 shared genes. The latter included calmodulin-binding transcription factor (*CAMTA*), known to be involved in male courtship song in *D. melanogaster* (Sato et al., 2019a) and associated with male song variation in crickets in the genus *Lapaula* (Xu & Shaw, 2021).

To test the effect of sympatry on intraspecific variation, we identified intraspecies interpopulation F_ST_ outliers by contrasting genetic differentiation between sympatric and allopatric populations for each species separately. Regions of increased differentiation were largely unshared between species (Figure 4E and G). For example, *T. commodus* contrasts identified 47 genes, and *T. oceanicus* contrasts identified 83, but only 4 were shared (Supplementary Table 4). Regions of increased absolute genetic divergence were similarly unshared between species. Between allopatric and sympatric *T. commodus* contrasts 37 genes were identified as outliers, and between *T. oceanicus* populations 49 genes were outliers, but only two were shared.

### Loci associated with intraspecific song and CHC variation

#### Male calling song

**Figure 5.**
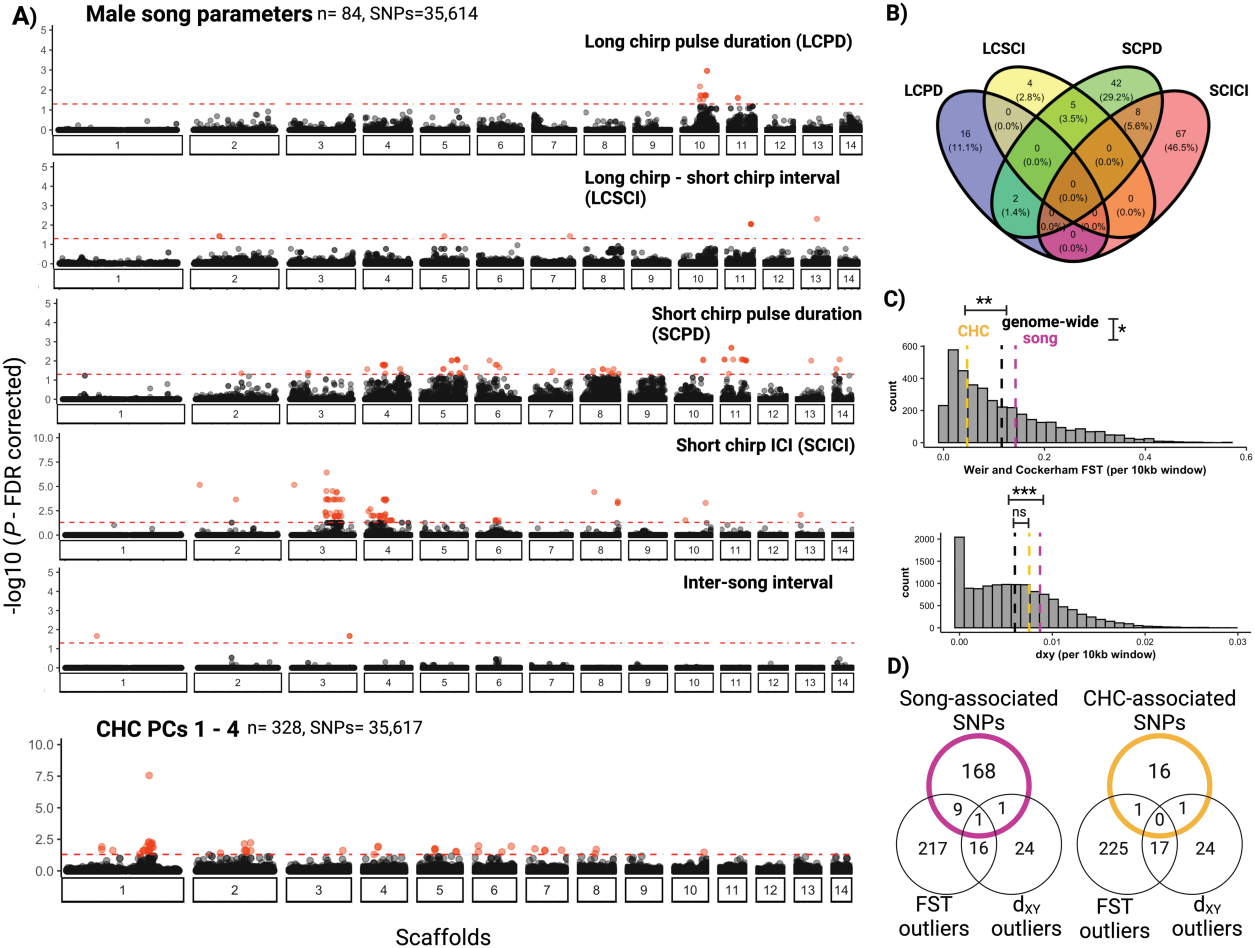
Linking intraspecific genetic variation in *T*. oceanicus for male calling song and CHCs with interspecific candidate barrier loci between *T. oceanicus* and *T. commodus.* (A) Significant genome-wide association (GWA) analyses. Only song parameters with at least one significant association are shown. The bottom panel shows GWA analysis for CHCs, combining sexes. The dotted red line and points indicate significance threshold (FDR-corrected *p* < 0.05) and associated SNPs, respectively. (B) The Venn diagram shows an overlap of male song trait SNPs. (C) Histograms of F_ST_ and d_XY_ calculated in 10 kb windows. Dotted pink and orange lines indicate mean F_ST_ and d_XY_ for 10 kb regions containing at least one SNP statistically associated with song and CHC variation, respectively. The dotted black line indicates genome-wide mean F_ST_ and d_XY._ Stars indicate level of significance for two-sample *t*-tests: 0.0001 = ***, 0.001 = **, 0.01 = *, 0.05 = . , >0.05 = ns. (D) Overlap of genes within F_ST_ and d_XY_ outlier regions (99th quantile of distributions) and song and CHC-associated genes.

Male advertisement song differs between *T. oceanicus* and *T. commodus* and is a primary barrier to gene flow between them (Hill et al., 1972; Bailey & Macleod, 2014; Hoy et al., 1977; Moran et al., 2020). Five of the 14 male calling song traits that we measured in F3 individuals from a *T. oceanicus* mapping population showed significant genomic associations in genome-wide association (GWA) tests. The traits were long-chirp pulse duration, the duration of long chirp-short chirp intervals, short-chirp pulse duration, short-chirp inter-chirp interval, and inter-song interval; in all cases, associated SNPs were distributed over more than one chromosome (Figure 5A). There was negligible evidence for genomic co-localization of different song traits, highlighting the complex architecture of male calling songs (see also Supplementary Figure 4) (Figure 5B). Overall, we found 162 significantly associated SNPs (corrected *p* < 0.05) (Supplementary Table 7), and these overlapped or were within 10 kb of 179 genes, which included orthologs in *D. melanogaster* that modulate courtship behavior (*takeout* and *lola*) (Dauwalder et al., 2002; Dinges et al., 2017; Lazareva et al., 2007; Sato et al., 2019).

#### Cuticular hydrocarbons

CHCs are long-chain waxy molecules that play a role in desiccation resistance but are also a sexual signal under multivariate sexual selection in *T. oceanicus* (Thomas & Simmons, 2009, 2010). Using gas chromatography-mass spectrometry of CHC profiles in F3 individuals of a *T. oceanicus* mapping population, 26 individual compounds were identified (Pascoal et al., 2020). Here, we performed GWA analyses on PC axes summarizing variation across CHC profiles. Altogether, we found 50 SNPs (Wald test, corrected *p* < 0.05) associated with variation in CHC production across 328 individuals. These SNPs overlapped or were within 10 kb of 22 genes (Supplementary Table 5), but none of the genes have previously been implicated in CHC variation or production. Consistent with previous analyses (Pascoal et al., 2020), we find a large peak of association for CHC production on the X chromosome (scaffold 1). Of the 50 significantly associated SNPs, 20 span a 4.2 MB region on the X chromosome containing *doublesex,* a candidate region implicated in a male wing-feminizing adaptation to an acoustically orienting parasite in Hawaiian populations of *T. oceanicus* (Figure 5A; bottom panel) (Zhang et al., 2021). When searching for sex-specific effects of CHC production, we found no associations after correcting for multiple testing. Follow-up analyses of the three peaks (peaks 4, 8, and 9) with the highest standard deviation among individuals indicated a similar pattern, albeit implicating different, peak-specific loci. For peaks 4 and 8, we found no significant hits. For peak 9, we found 25 significant hits, all on chromosome 5, which did not overlap with any of our hits using the PC axes (Supplementary Figure 8). Five genes overlapped with these 25 hits, but as with the PC-based analysis, none of these genes were functionally annotated as known CHC biosynthesis genes.

#### GEA tests

In total, we identified 2,418 SNPs associated (*p* < 0.001) with latitude and 1,831 associated (*p* < 0.001) with longitude. Only 867 SNPs (*p* < 0.001) were significantly associated with both latitudinal and longitudinal gradients across the 16 populations sampled. These SNPs corresponded to 184 genes with annotations. We asked whether any of the song or CHC-associated genes showed allele frequency changes associated with latitudinal and longitudinal variables. We found only four environment-associated genes, which also showed significant song-associations (out of 179 song-associated genes). These genes include *seven-up* and *methuselah-like 2* (mthl2). Similarly, only two CHC-associated (out of 22) genes showed significant associations with both latitudinal and longitudinal variables.

### Association of intraspecific sexual trait loci with interspecific divergence

To identify genes simultaneously involved in intraspecific male courtship song and CHC variation as well as interspecific divergence, we compared F_ST_ and d_XY_ in 10kb windows containing SNPs (and presumably gene regions) associated with song and CHC variation with background levels of genomic divergence between *T. oceanicus* and *T. commodus*. Whilst song genes showed significantly higher genomic differentiation and divergence compared to the genetic background (two-sample *t*-tests: F_ST_, *t *= −2.16, *p* = 0.036; d_XY_, *t *= −4.31, *p* < 0.0001)(Figure 5C), genes associated with intraspecific CHC variation showed significantly lower differentiation but comparable genomic divergence to the genetic background (two-sample t-test: F_ST_, *t *= 3.15, *p* = 0.005; d_XY,_*t* = −1.87, *p* = 0.06) (Figure 5C). Additionally, we tested for evidence of stronger diversifying selection of song and CHC-associated genes in sympatry compared to allopatry and found no difference in F_ST_ between song-associated (Wilcox test; *W* = 1712, *p* = 0.9) and CHC-associated (Wilcox test; W = 279, *p *= 0.3) genes in sympatry compared to allopatry. Altogether, our results imply stronger interspecific divergence of song-associated gene regions compared to CHC-associated gene regions.

Next, we examined which genes in each genome-wide associated test also showed evidence consistent with relatively extreme levels of interspecific genomic divergence (Figure 5D). For CHC variation, one gene showed a signal of high genomic differentiation (*phtf*), and one gene showed a signal of high genomic divergence (*RYK*), providing little evidence that genes associated with intraspecific CHC variation are involved in interspecific divergence. In contrast, we found 10 intraspecific song-associated genes with high genetic differentiation between *T. commodus* and *T. oceanicus*, and 2 genes (*semphaorin-2A* and *Syntrophin-like 2*) also showing signals of high genomic divergence. The gene *semaphorin-2A* (SEM2A) is significantly associated with short-chirp inter-chirp interval (Scaffold 4, position: 44,431,198; Wald test, uncorrected *p *< 0.0001 and corrected *p* = 0.01) and shows elevated signals of differentiation (F_ST_ = 0.53) and divergence (d_XY_ = 0.02).

To test if song genes were under recent positive selection in *T. commodus* and *T. oceanicus*, we focused on scaffolds 3 and 4, where large genomic regions show consistent, significant associations with the short-chirp inter-chirp interval. Within those focused regions and globally across scaffolds 3 and 4, we calculated species-specific integrated haplotype scores (iHS) and asked which song-associated genes, if any, showed signatures of selective sweeps. Examining the most extreme values of the haplotype statistic (iHS—99th percentile), we found no scaffold three genes that met our conservative iHS cutoff and were significantly associated with any song parameter in *T. oceanicus*. Only one gene (*Tudor-SN*) associated with short-chirp inter-chirp intervals showed signals consistent with a selective sweep in *T. oceanicus* (iHS = 3.31, log *P =* 3.03). For scaffold 4, we similarly found no overlap between iHS outliers and song-associated genes in *T. commodus*, but we found six genes that met both criteria in *T. oceanicus*, including *semaphorin 2A*, *seven-up,* and *Syntrophin-like 2*.

## Discussion

Empirical tests that link the genetics of intraspecific variation in mating traits with the genetics of reproductive isolation between species are rare despite their importance for testing seminal models of speciation by sexual selection (Kopp et al., 2017). Our analysis of Australian field cricket sister species provides a rare opportunity to explore the link between microevolutionary processes and macroevolutionary pattern. Overall, through a combination of intraspecific GWA tests, gene ontology, interspecific population genomic scans of divergence, and explicit tests for positive selection, we recovered a few genes underlying sexual traits within *T. oceanicus* that were also implicated as putative barrier loci between *T. oceanicus* and *T. commodus*. However, the few that we did identify showed evidence of divergent selection that we cannot readily attribute to natural selection. We discuss the implications and limitations of these findings and identify areas where specific experimental advances will be required to overcome common obstacles that impede understanding of the genomics of speciation via sexual selection.

Our study demonstrates that *T. oceanicus* and *T. commodus* are genetically distinct and that they are likely to hybridize only rarely in contemporary populations. Nevertheless, these sister species show striking patterns of heightened genome-wide differentiation in regions of sympatry despite evidence of elevated levels of historical gene flow in sympatry compared to allopatry. Such genomic signals are consistent with reproductive character displacement in sympatry (Garner et al., 2018), perhaps suggesting a role for reinforcement in driving divergence between *T. oceanicus* and *T. commodus*. Patterns of increased differentiation in sympatry may also be explained by demographic shifts specific to sympatric populations of *T. commodus*, for which we also find evidence (Supplementary Figure 6). Loci associated with intraspecific variation in male advertisement songs and CHC profiles did not collectively show evidence of divergent selection between species, but the small number may play a disproportionate role in species divergence. Intriguingly, the latter was mostly limited to loci associated with advertisement song variation within *T. oceanicus* but less so CHCs, suggesting co-option of the genetic architecture of intraspecific mating signals depends on the presence or absence of constraints imposed by countervailing sources of selection. It is, however, difficult to disentangle whether the genomic divergence of candidate loci identified here occurred before the split or after the split of *T. commodus* and *T. oceanicus*. Additionally, it is also unclear how much of the genomic landscape observed in this study, particularly in genomic regions containing interesting candidate loci, can be explained by linked selection and other unconsidered genomic features (Ravinet et al., 2017). Application of ancestral recombination graph and model-based approaches on whole-genome resequencing for both species may help to clarify the specific role of candidate loci identified here (Hejase et al., 2020; Laetsch et al., 2023).

So far, only a few studies have investigated whether genome-wide patterns of differentiation reflect a signal of reinforcement (Hopkins et al., 2012; Smadja et al., 2015). We found evidence of significant, increased differentiation attributable to divergence in sympatric *T. commodus* populations. Distinguishing between scenarios that may explain the patterns observed here is challenging. For instance, whilst we have shown evidence of a uniform increase in genetic differentiation in sympatry, it is unclear whether reinforcement drove this signal (Hoskin & Higgie, 2010; Noor, 1999). For instance, nucleotide diversity appears to be lower in sympatric populations of *T. commodus*, which may enhance signals of differentiation in the contact zone (Supplementary Figure 6). Additionally, the phenotypic divergence of male advertisement song and CHC profiles between *T. oceanicus* and *T. commodus* do not seem to be strongly enhanced in the contact zone (Moran et al., 2020). Both *T. oceanicus* and *T. commodus* are collected in the same localities and occupy similar ecological niche spaces, suggesting local adaptation and ecological character displacement does not best explain the patterns observed here. Using GEA tests, we demonstrate that there is little overlap that we could detect between genomic loci associated with sexual traits and genes potentially under local adaptation, indicating little support for the role of ecology in driving the divergence of mating traits.

For the five song parameters that showed significant genetic associations, we show that their genetic bases are largely polygenic (Figure 4A and B). Such genetic architecture is consistent with expectations of a complex genetic basis of male courtship song in other insects such as *Drosophila* (Gleason & Ritchie, 2004; Pischedda et al., 2014; Turner et al., 2013). Candidate barrier loci were dominated by genes enriched for neurological functions, indicating potential behavioral rewiring and several of the small number of genes enriched in both intraspecific and interspecific analyses have been implicated in the control of courtship song variation in other species (Blankers et al., 2018; Sato et al., 2019; Turner et al., 2013; Xu & Shaw, 2019). For example, genes identified in our outlier scans may also play a role in species divergence in rapid radiation of endemic Hawaiian crickets, and several have been reliably annotated as *D. melanogaster* orthologs and were previously implicated in *Drosophila* courtship behavior (Supplementary Table 6). *Lola* is one such promising candidate. In *Drosophila*, it is involved in neuronal reprogramming that interacts with *fruitless*, a regulator of courtship circuitry. It is associated with long-chirp pulse duration in *T. oceanicus* in this study (corrected *p*: 0.018) and is also under a moderate-effect QTL for mating song rhythm variation in *Laupala* (Blankers et al., 2018; Dinges et al., 2017; Sato et al., 2019b). Some of the song-associated genes found in our GWA tests were also outliers in our F_ST_ and d_XY_ genome scans and represent additional candidates for further study. For example, *Syn2* is associated with variation in short-chirp inter-chirp intervals and shows evidence of positive selection in this study via extended haplotype homozygosity tests, and *Syn1* in *Drosophila melanogaster* has been associated with variation in inter-pulse intervals of courtship song through an evolve-and-resequence experiment (Turner et al., 2013). Although we detect promising candidate loci, the use of reduced representation sequencing in GWA analyses here and sample size constraints might have potentially resulted in other important loci as well as small-effect loci being missed altogether.

We recapitulate findings from Pascoal et al. (2020) and show that close to half the associated SNPs found in our GWA for CHC production are found in a 20 Mb region around *doublesex* (Pascoal et al., 2014, 2020). However, only two SNPs associated with CHC production are found within or around genes that are also outliers in our genome scans, and genes associated with intraspecific CHC production are significantly less differentiated compared to the genomic background (Figure 5C), suggesting they may be under strong selective constraint between species. In *Teleogryllus*, CHC profiles are heritable (Thomas & Simmons, 2008), subject to both natural and sexual selection (Berson et al., 2019; Mitchell et al., 2023; Steiger et al., 2015) and are likely under multivariate sexual selection (Thomas & Simmons, 2009, 2010). CHCs are implicated in species divergence in the European crickets *Gryllus bimaculatus* and *G. campestris* (Tyler et al., 2015). Whilst we do not explicitly assay CHC profiles in a mate-choice context, candidate genes for CHCs identified here may potentially be implicated in species divergence. In contrast with song-associated genes, patterns of genetic variation in CHC-associated genes and underlying phenotypic variation may be mediated by natural and sexual selection acting in opposing directions, which is a central prediction for classic models of sexual selection (Fisher, 1915, 1958; Lande, 1981; Kirkpatrick, 1982; see also Berson et al., 2019). However, although dimensionality reduction capture collinearity between cuticular hydrocarbons, they may obscure genetic variation associated with singular peaks in CHC profiles (Dembeck et al., 2015; Martin & Drijfhout, 2009). More detailed association and functional analyses are required to identify the full complement of large- and moderate-effect loci involved in CHC biosynthesis.

## Conclusions

The evolution of divergent mating traits is an important mechanism that can restrict gene flow between nascent lineages and maintain species boundaries during species coexistence. Our results suggest that the genetic architecture of intraspecific variation in sexual traits can be co-opted during species divergence but that this co-option is subject to important limitations. In *T. oceanicus*, such co-option is likely driven by a limited subset of loci out of a wider set controlling highly polygenic acoustic signaling traits. Chemical signals are similarly polygenic but are perhaps under considerable ecological constraint and therefore not as important for divergence between *T. commodus* and *T. oceanicus*.

Our study represents one of few dual assessments of the genetic basis of intraspecific mating traits and divergent interspecific selection in natural populations. The findings support a scenario where few but essential loci play a dual role in intraspecific sexual selection and interspecific divergence, whereas many other loci contribute to either process but do not mechanistically link the two. The quest to connect the microevolutionary process and macroevolutionary pattern during speciation by sexual selection might, therefore, profit from combinatorial approaches such as those we present here to narrow the focus on rare “speciation-and-sexual-selection” genes.

## Supplementary material

Supplementary material is available online at *Evolution Letters*.

qrae042_suppl_Supplementary_Tables

qrae042_suppl_Supplementary_Figures

## Data Availability

Raw sequence data used in this paper can be found in NCBI under the BioProject IDs: PRJEB26502 (population genomics) and PRJEB24786 (genotype–phenotype mapping). Male calling song and CHC data are available as Supplementary Material and archived in Dryad (https://doi.org/10.5061/dryad.g4f4qrfzt).
