# Supplementary material for: Testing the genomic overlap between intraspecific mating traits and interspecific mating barriers
Source: Evol Lett. 2024 Aug 14;8(6):902–15. doi: 10.1093/evlett/qrae042 (PMC11637687; doi:10.1093/evlett/qrae042)
Supplement: qrae042_suppl_Supplementary_Figures [file qrae042_suppl_supplementary_figures.docx]

# Supplementary figures


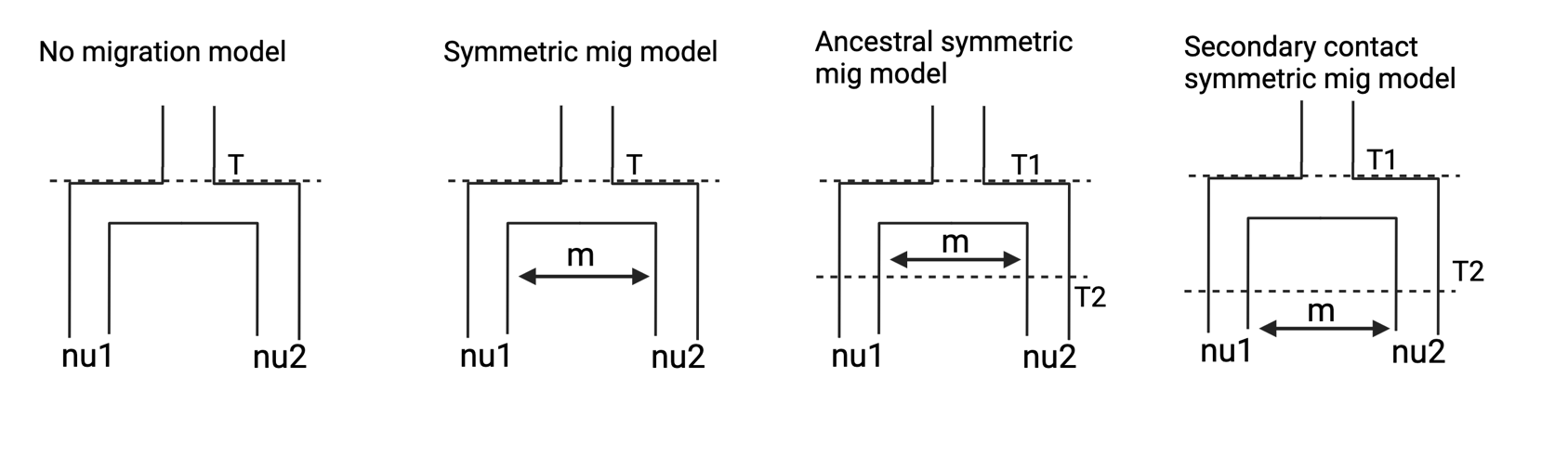


**Supplementary figure 1:** Illustration of demographic models tested. T refers to divergence times. T1 indicates the initial divergence of species. Migration is denoted by lines with arrows and letter “m’, and effective population sizes are denoted by “nu”. Effective population sizes are further denoted by either ‘a’ or ‘b’ in expansion models.


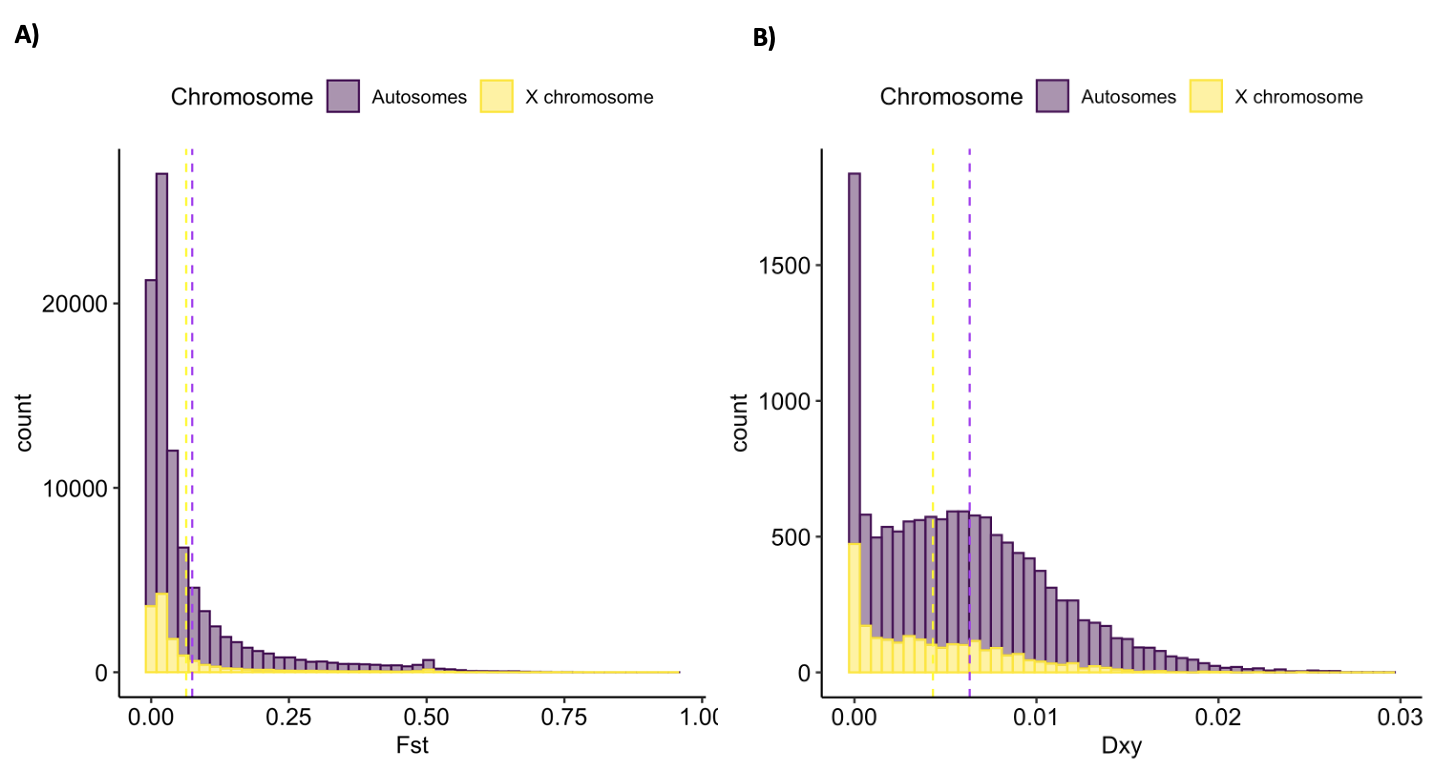


**Supplementary figure 2:** Histograms showing autosomal and X chromosome **(A)** genetic differentiation (F_ST_) and **(B)** divergence (d_XY_). Dashed lines indicate mean F_ST_ and d_XY_ for the autosomes and X chromosome across the 14 longest scaffolds.


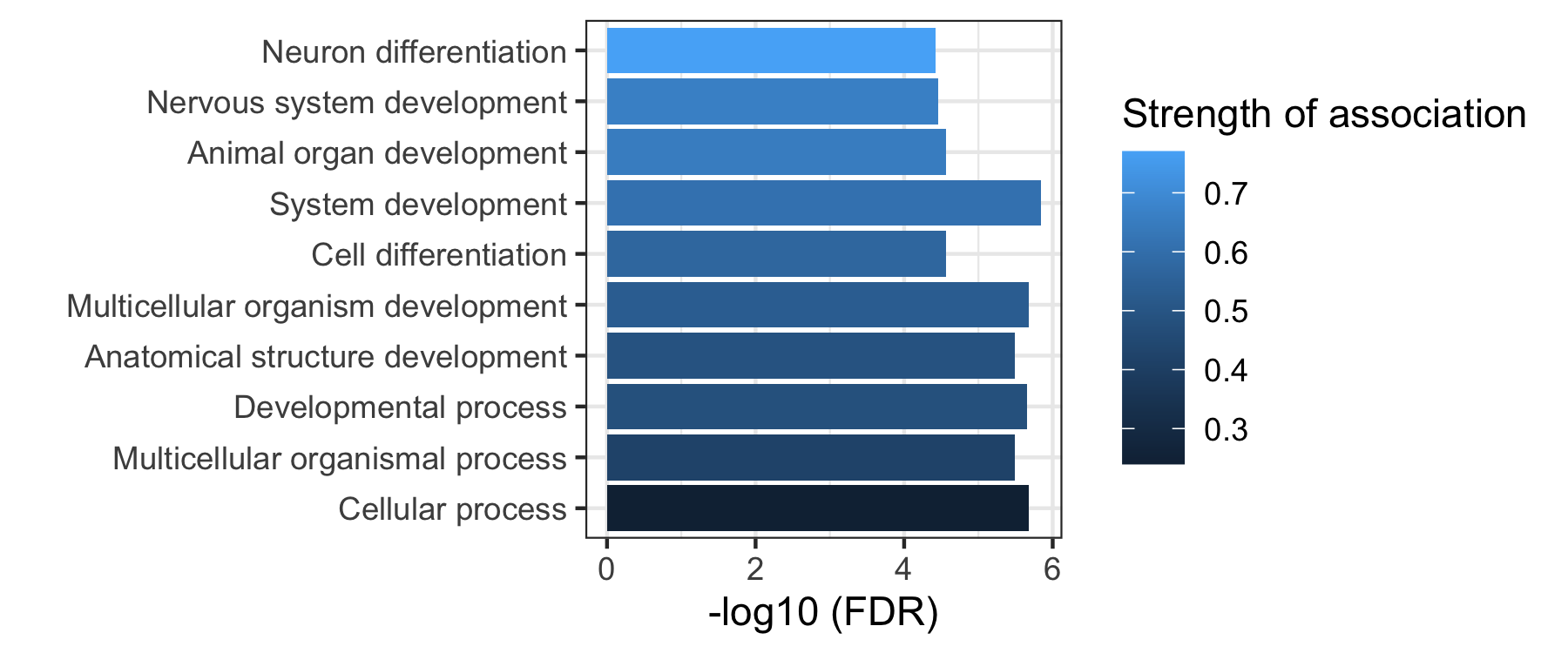


**Supplementary Figure 3:** Gene ontology for biological processes on genes with at least one FST outlier (99^th^ quartile) SNP.


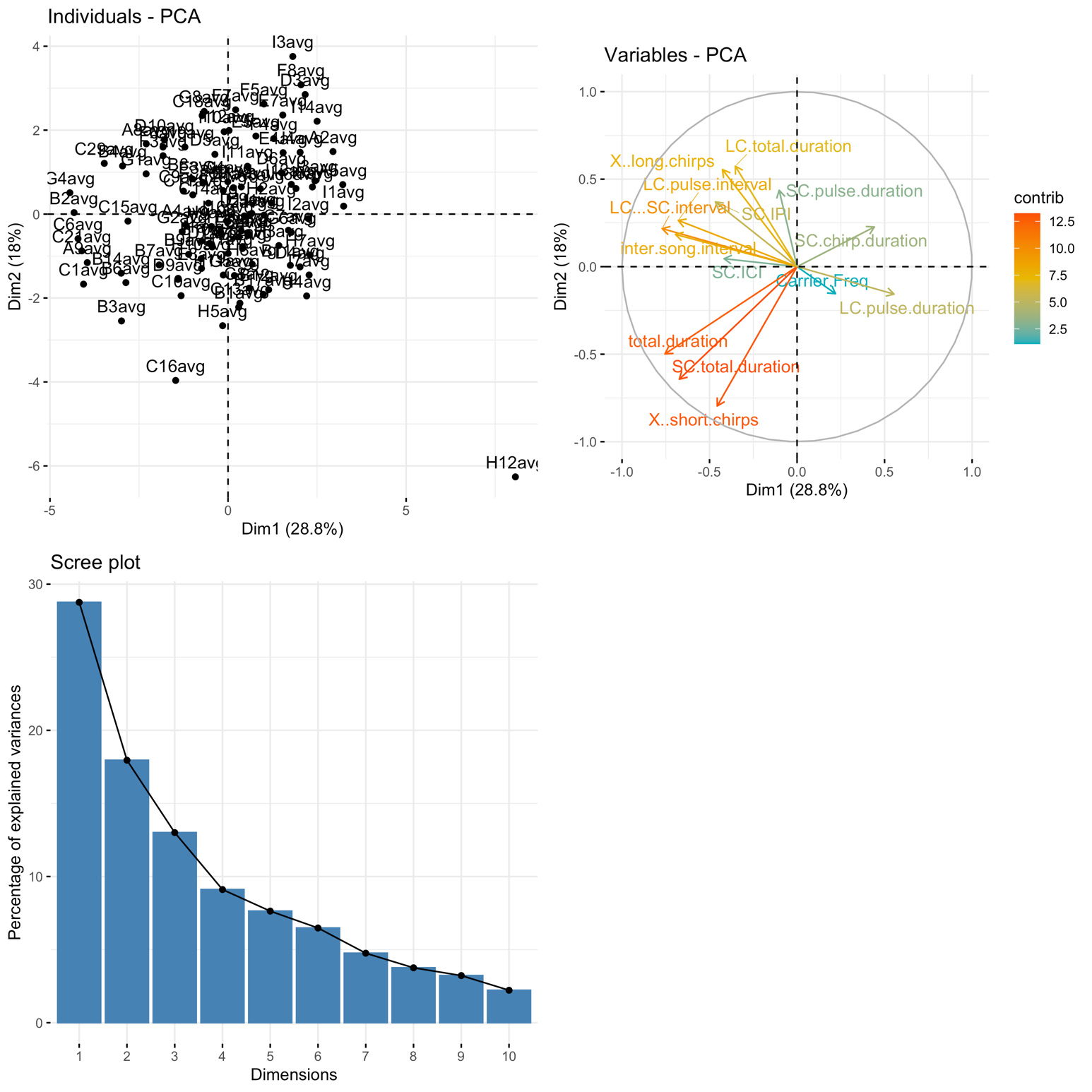


**Supplementary Figure 4:** Principal components analysis of male advertisement song. A) Showing individuals from the F3 mapping population across PC 1 (explaining 28.8% of variation in male advertisement song) and PC2 (explaining 18%). B) Showing the relationship between male advertisement song parameters and PC1 and PC2. C) Proportion of variance explained by each PC from PC1 – PC10.

**A)**

**C)**

**B)**


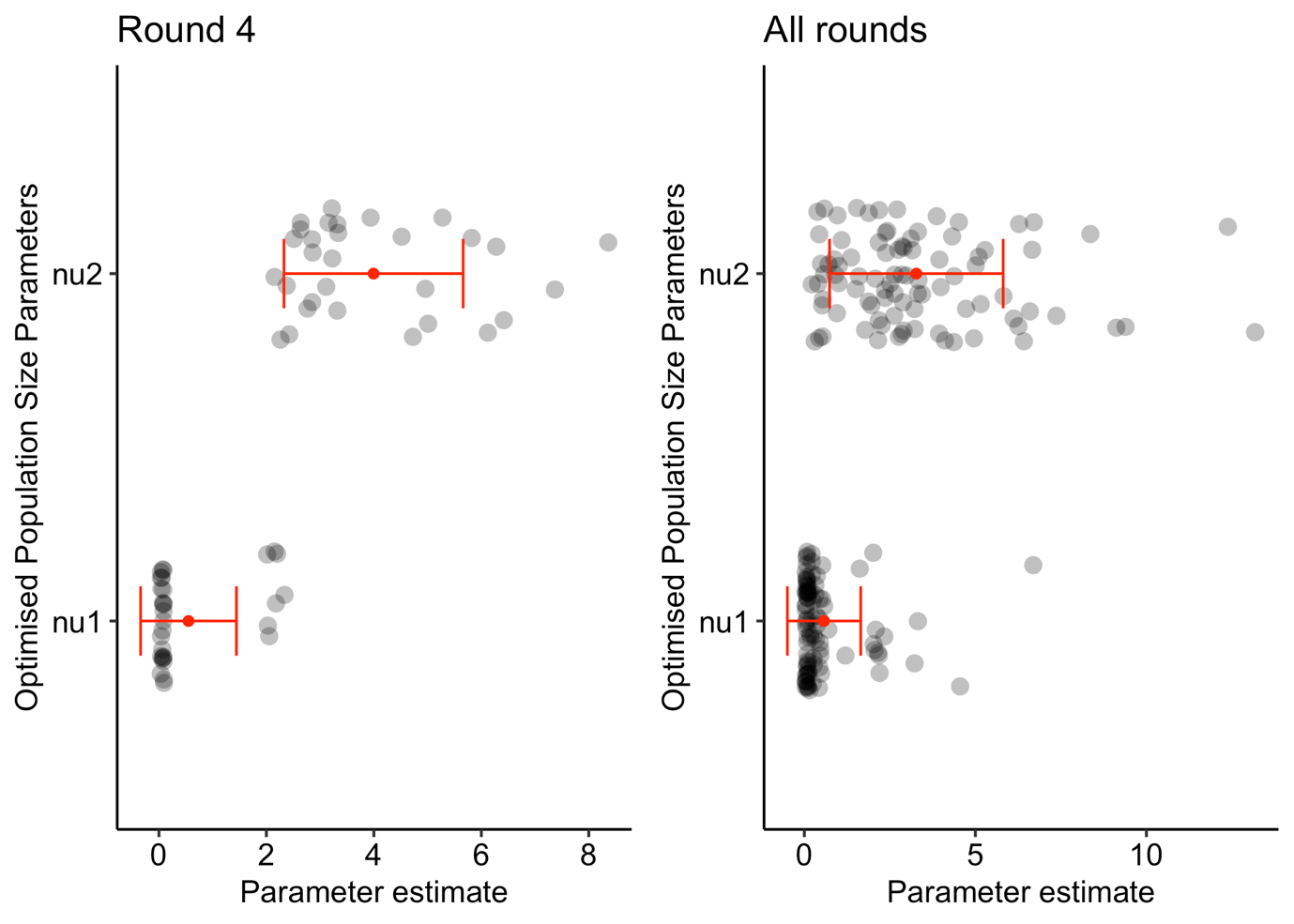


**Supplementary Figure 5:** Optimised parameter estimates for population size for *T. commodus* (nu1) and *T. oceanicus* (nu2) for the final round of parameter optimization in *dadi* compared to all rounds. Dots indicate parameter estimate for each model replicate and red bars are standard error of parameter estimates for round 4 and across all rounds.

*


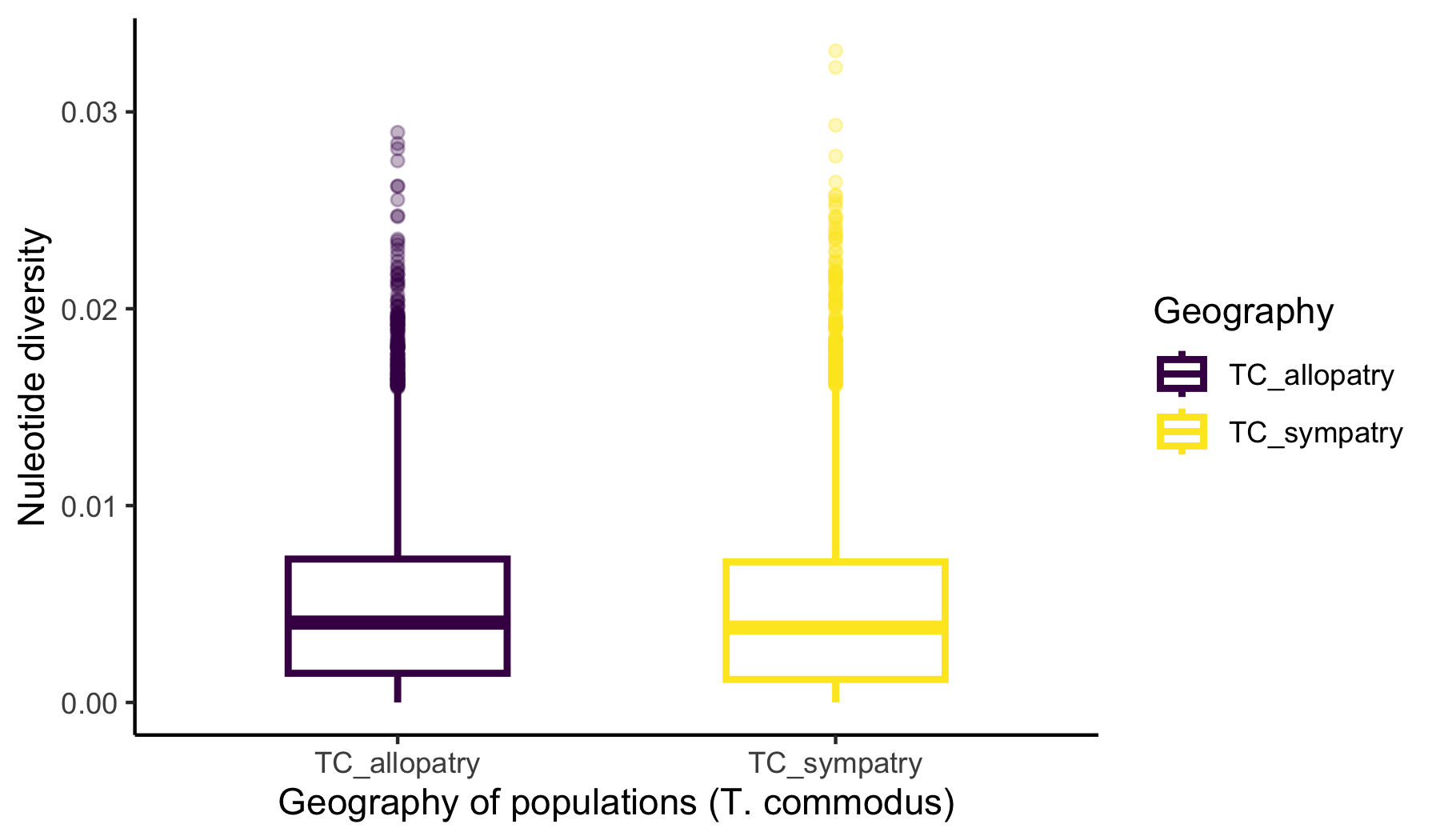


**Supplementary Figure 6:** Nucleotide diversity calculated for *T. commodus* populations in allopatry and sympatry (with *T. oceanicus*). Stars indicate level of significance for two-sample t-tests: 0.0001 = ***, 0.001 = **, 0.01 = *, 0.05 = . , >0.05 = ns.

**
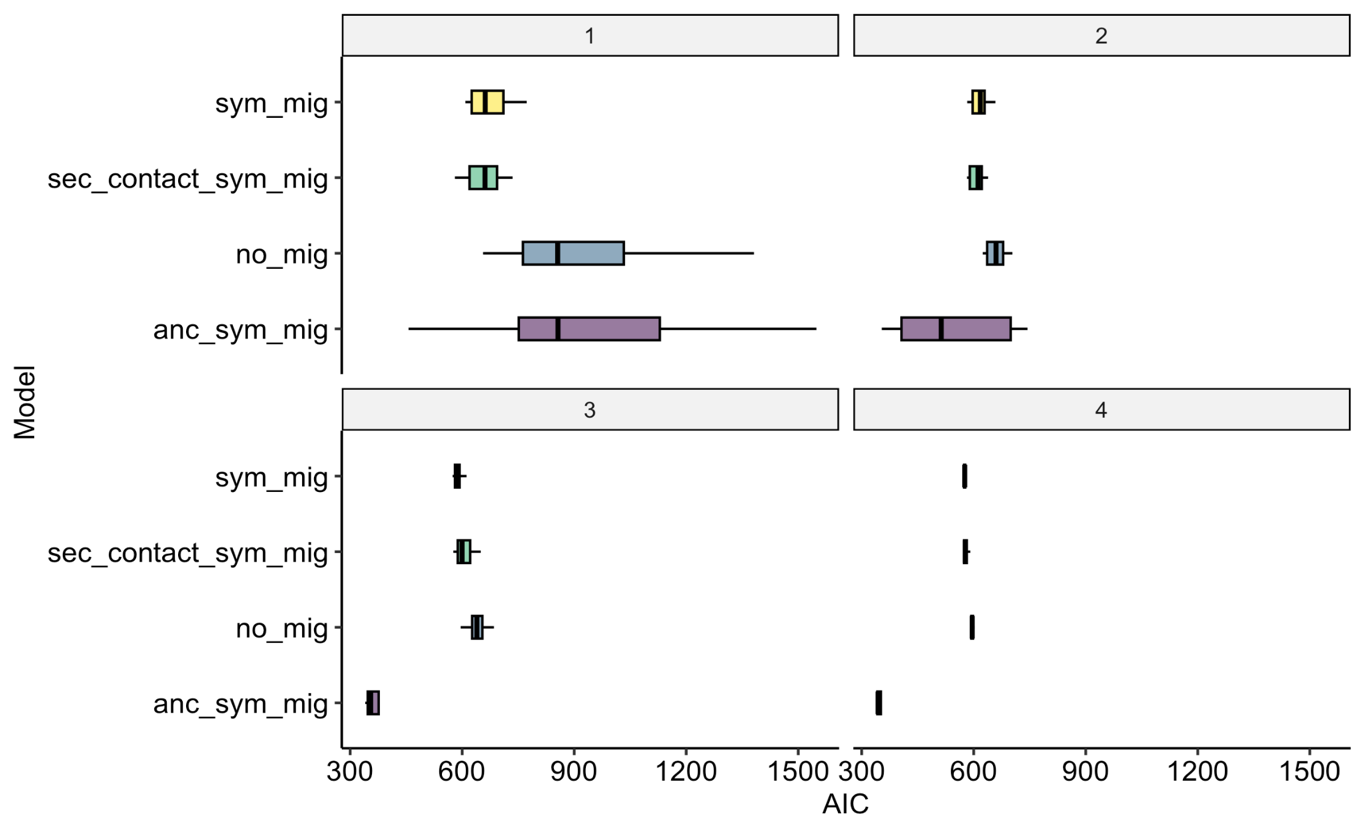
**

**Supplementary Figure 7:** Demographic model fitting by optimization round. Model names and parameter estimates for each model can be found in Supplementary figure 1. AIC is used to explore model selection. Default starting parameters for each model in the first round are randomized. The best-fitting parameter estimates for each model are then used as starting parameters for the next round.


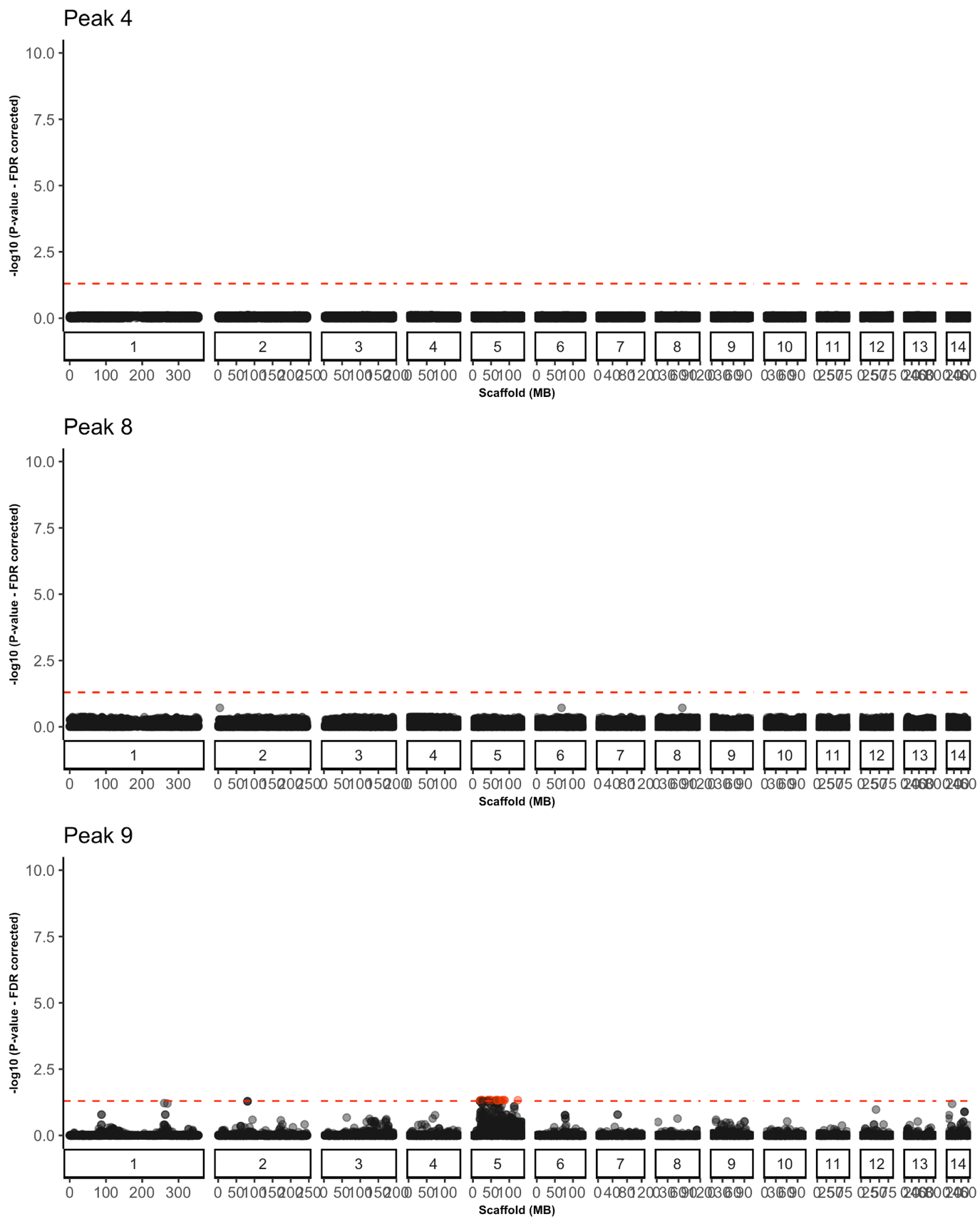


**
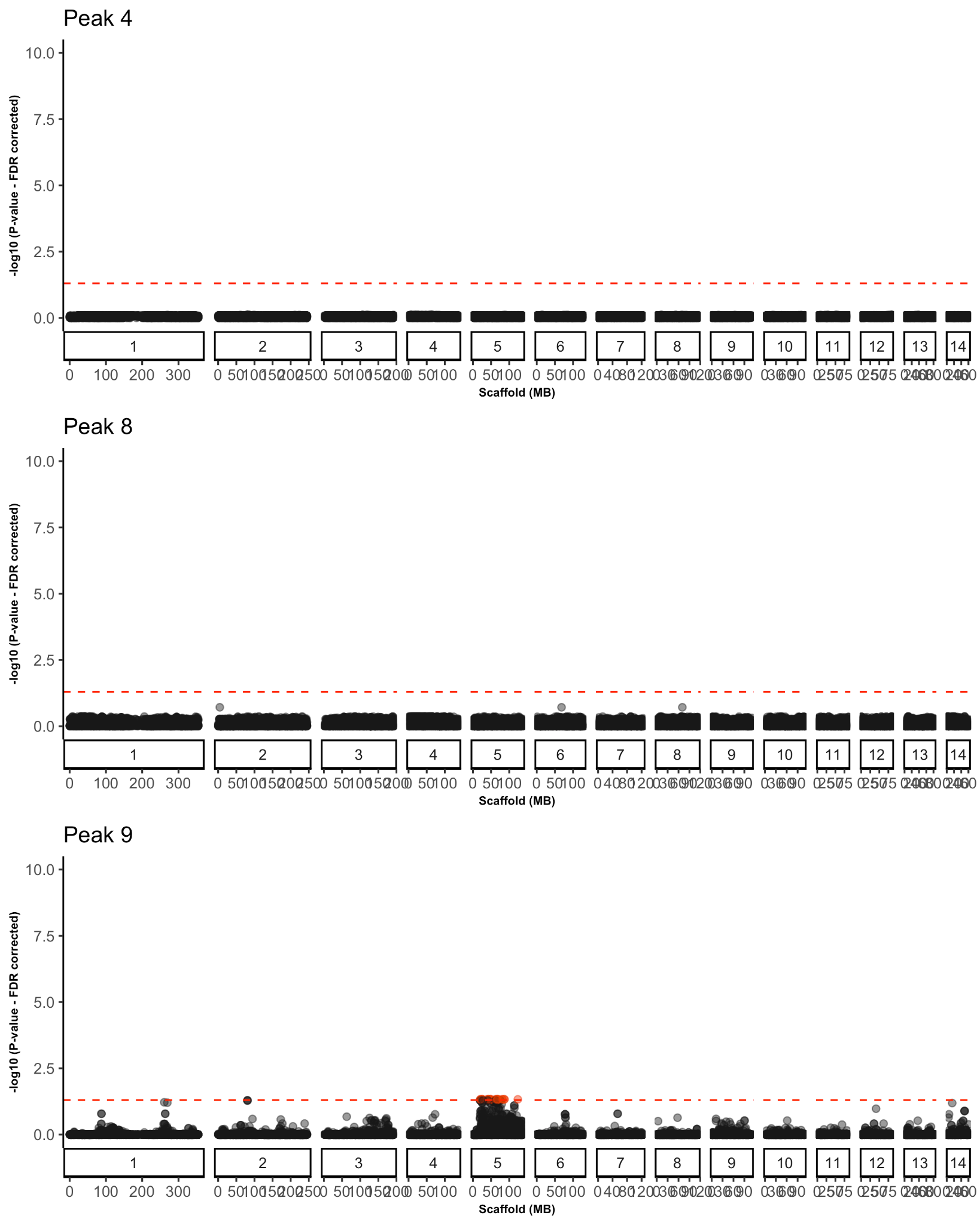
**

**
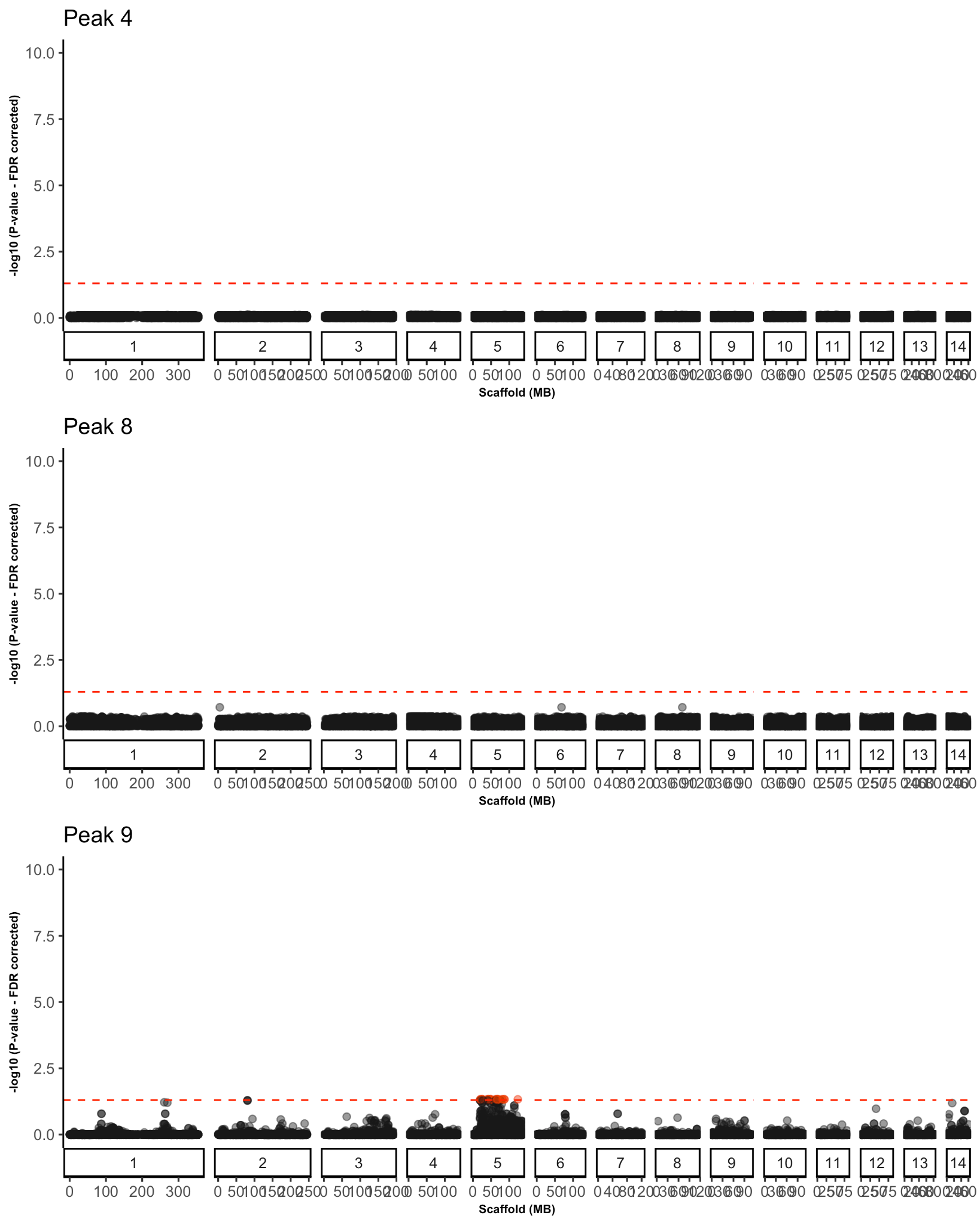
**

**
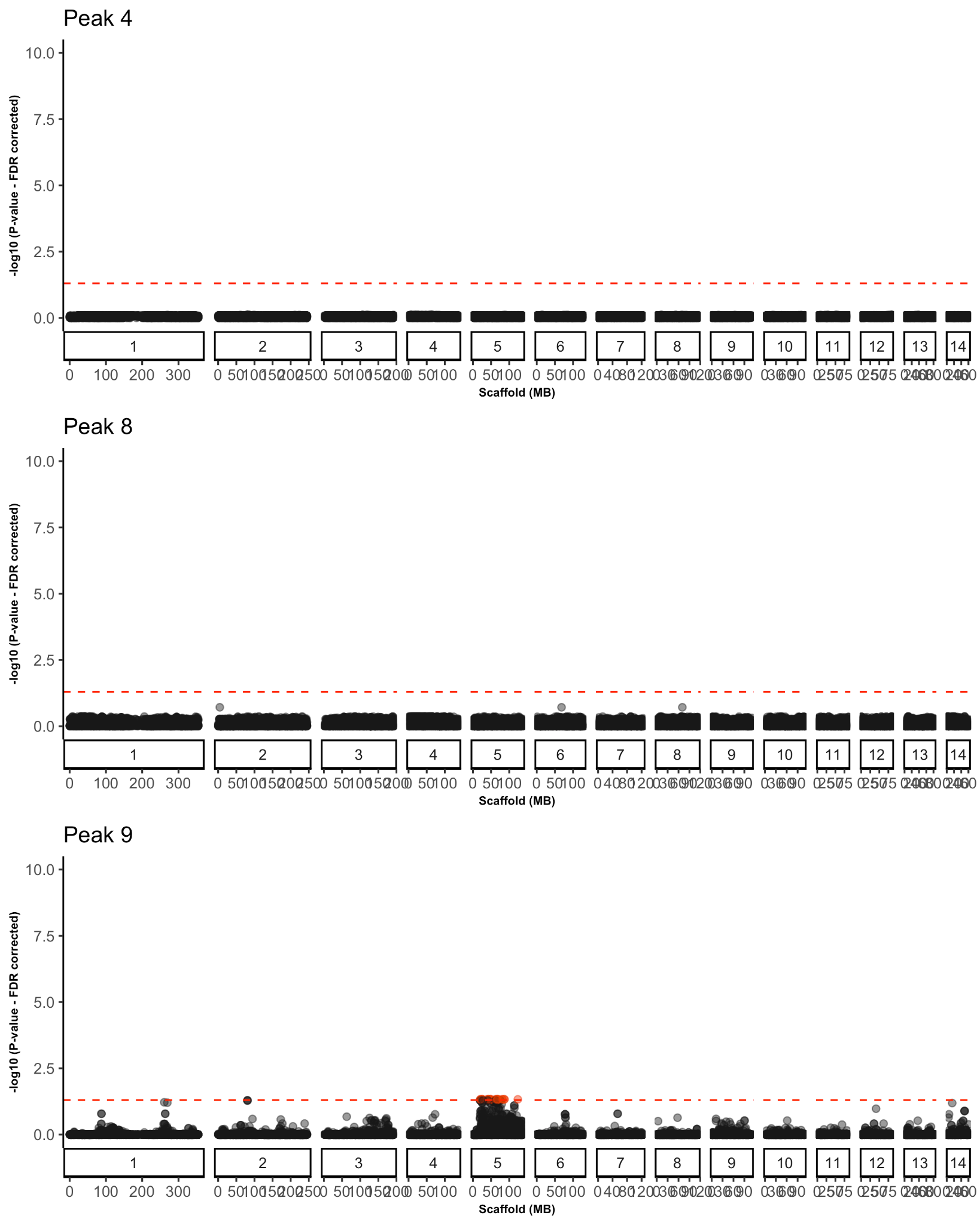
**

**Supplementary Figure 8:** Genome-wide association analyses using three CHC peaks with most variation between individuals. All three analyses use all individuals of both sexes. Dotted red line and red points indicate significant thresholds and SNPs with significant associations (FDR < 0.05).
